# MiR-29b antagonizes the pro-inflammatory tumor-promoting activity of multiple myeloma-educated dendritic cells

**DOI:** 10.1038/leu.2017.336

**Published:** 2018-01-30

**Authors:** C Botta, M Cucè, M R Pitari, D Caracciolo, A Gullà, E Morelli, C Riillo, L Biamonte, M E Gallo Cantafio, R Prabhala, C Mignogna, A Di Vito, E Altomare, N Amodio, M T Di Martino, P Correale, M Rossi, A Giordano, N C Munshi, P Tagliaferri, P Tassone

**Affiliations:** 1 Department of Experimental and Clinical Medicine, Magna Graecia University, Catanzaro, Italy; 2 Veterans Administration Boston Healthcare System, West Roxbury, MA, USA; 3 Department of Health Science, Magna Graecia University, Catanzaro, Italy; 4 Department of Medical Oncology, ‘Bianchi-Melacrino-Morelli’ Hospital, Reggio Calabria, Italy; 5 Department of Human Pathology and Oncology, University of Siena, Siena, Italy; 6 Department of Biology, Center for Biotechnology, Sbarro Institute for Cancer Research and Molecular Medicine, College of Science and Technology, Temple University, Philadelphia, PA, USA; 7 Department of Medical Oncology, Dana-Farber Cancer Institute, Harvard Medical School, Boston, MA, USA

## Abstract

Dendritic cells (DCs) have a key role in regulating tumor immunity, tumor cell growth and drug resistance. We hypothesized that multiple myeloma (MM) cells might recruit and reprogram DCs to a tumor-permissive phenotype by changes within their microRNA (miRNA) network. By analyzing six different miRNA-profiling data sets, miR-29b was identified as the only miRNA upregulated in normal mature DCs and significantly downregulated in tumor-associated DCs. This finding was validated in primary DCs co-cultured *in vitro* with MM cell lines and in primary bone marrow DCs from MM patients. In DCs co-cultured with MM cells, enforced expression of miR-29b counteracted pro-inflammatory pathways, including signal transducer and activator of transcription 3 and nuclear factor-κB, and cytokine/chemokine signaling networks, which correlated with patients’ adverse prognosis and development of bone disease. Moreover, miR-29b downregulated interleukin-23 *in vitro* and in the SCID-*synth-hu in vivo* model, and antagonized a Th17 inflammatory response. All together, these effects translated into strong anti-proliferative activity and reduction of genomic instability of MM cells. Our study demonstrates that MM reprograms the DCs functional phenotype by downregulating miR-29b whose reconstitution impairs DCs ability to sustain MM cell growth and survival. These results underscore miR-29b as an innovative and attractive candidate for miRNA-based immune therapy of MM.

## Introduction

Multiple myeloma (MM) is an incurable malignancy characterized by uncontrolled clonal proliferation of malignant plasma cells (PCs) within the bone marrow (BM). Although novel therapeutic strategies have recently improved the clinical outcome, patients invariably still progress to a drug-resistant disease.^1^

It is well known that a crucial cross-talk between tumor cells and ancillary cell components takes place within the human BM microenvironment (huBMM). This complex network of interactions promotes MM progression and drug resistance, neo-angiogenesis, bone destruction and immune escape.^2, 3, 4, 5^ Specifically, an inflammatory/immune-suppressive *milieu*, which may account for the failure of immunotherapy in MM, has been recently described.^4, 5, 6, 7^ Among several components of the huBMM, cells expressing CD28 ligands CD80 and CD86, including macrophages and dendritic cells (DCs), have been found to induce melphalan and bortezomib resistance through the activation of the CD28/PI3K/AKT signaling in MM cells.^4^ DCs, which are potent antigen-presenting cells, with a crucial role for the ignition of both innate and adaptive immune response and for maintaining immunological tolerance,^8^ have been suggested as pivotal elements in modulating response to therapeutics.^2, 8, 9^ Depending on their maturation and phenotype, as well as on microenvironment cell-to-cell interaction and/or soluble mediators, DCs orchestrate both activation of immune response and tolerance by inducing the polarization of T-helper lymphocytes into different functional T-cell subpopulations, such as Th1, Th2, Th17 and regulatory T cells.^10^ Different studies described extensive DCs infiltration (up to 10%) in BM from MM patients.^11, 12^ DCs are attracted in the MM-induced inflammatory huBMM and reprogrammed: (i) to directly sustain MM cells growth;^11^ (ii) to differentiate in osteoclast-like cells;^13^ (iii) to expand regulatory T cells,^14^ and (iv) to induce Th17 cells, thus enhancing the inflammatory huBMM and promoting bone disease^15^ through different mechanisms.^16, 17^ It is now a common view that MM cells enforce surrounding cells to produce a tumor permissive huBMM. It is noteworthy that reprogramming the immune response requires rapid changes at both transcriptional and posttranscriptional level, and microRNAs (miRNAs) are likely to have a role in regulating such events.

miRNAs are small non-coding RNA molecules that regulate gene expression at posttranscriptional level.^18^ Each miRNA may target up to hundreds of different transcripts, thus influencing multiple biological pathways including those relevant in cancer and immune response.^2, 19^ Currently, several miRNAs have been found to be deregulated in MM^20, 21, 22, 23, 24^ or in huBMM cells^25, 26^ and such deregulation is considered to have a key role in MM pathogenesis. Moreover, different studies demonstrated that miRNAs are essential regulators of DCs differentiation and functions.^27^

On this basis, we investigated whether (i) changes in the miRNA network occur in MM-associated DCs, (ii) these changes reprogram DCs to produce a tumor-permissive huBMM and (iii) interference within the DCs’ miRNA network may offer a novel therapeutic opportunity for MM.

## Materials and methods

### Microarray data sets

In order to evaluate the differentially expressed miRNAs in mature DCs (mDCs) and tumor-associated DCs (TA-DCs) compared with immature DCs (iDCs) we searched the Gene Expression Omnibus (GEO) website (http://www.ncbi.nlm.nih.gov/geo/) and the web for public available data sets. All microarray data sets from GEO repository using human or murine specimen under the search terms ‘dendritic cells’ and ‘microRNA profiling’ as of July 2016 were reviewed. To be included in our analysis, microarray-based miRNAs expression profiles should have been performed on iDC and mDCs (different maturation protocols were allowed) or TA-DCs from both human and mice. Normalized data from GEODatasets, GEO2R or directly from published papers were used to perform the analysis.

### MM patient-derived cells, healthy donor-derived cells and cell lines

Peripheral blood mononuclear cells (PBMCs) and BM mononuclear cells were obtained by Ficoll-Hypaque (Lonza Group, Basel, Switzerland) gradient separation of buffy coats of heparinized blood samples collected from healthy adult donors or MM patients, who provided informed consent according to institutional IRB regulations. CD14+ monocytes and CD3+ lymphocytes were then isolated by immunomagnetic separation with CD3 and CD14 microbeads (from either Miltenyi Biotech, Gladbach, Germany, or StemCell Technologies Inc, Vancouver, BC, Canada, or BD Bioscience, San Jose, CA, USA). Purity of the sample was assessed through flow cytometry and was >95% in all experiments. MM cell lines were cultured as described elsewhere.^20^ CD11c^+^CD45^+^ DCs were sorted from BM mononuclear cells using BD FACSAria III cell sorter (BectonDickinson, Heidelberg, Germany).

### DC generation

DCs were generated from healthy donor (HD) monocytes as described elsewhere.^28^ Additional details are reported in the Supplementary Materials and Methods section.

### DC transfection

Synthetic miRNA mimics were purchased from Ambion (Applied Biosystems, Carlsbad, CA, USA). DCs (1 × 10^6^) were transfected with scrambled (miR-NC) or synthetic pre-miR-29b (miR-29b) at a final concentration of 100 nM, using Neon Transfection System (Invitrogen, Carlsbad, CA, USA), (1,250 V, 30 ms, 2 pulses). The protocol optimization led us to achieve a transfection efficiency of 60–70% with a mortality <25% at 24 h evaluated by flow cytometric analysis relative to a green fluorescent protein control plasmid associated to 7-aminoactinomycin D.

### Flow cytometry

DCs, lymphocytes and tumor cells were collected with trypsin-free EDTA (Sigma, Steinheim, Germany) 2 mMM solution, washed twice with phosphate-buffered saline (PBS) containing 0.5% bovine serum abumin and distributed into 3 ml tubes (10^6^ cell/tube). Fluorochrome-conjugated antibodies against CD14, CD83 (BD Bioscience) and CD3, CD11c, CD45, CD86 and B7H3 (Miltenyi Biotech) were used to label cells according to producer’s guidelines. Cells were then acquired by flow cytometry (FACSCanto II, Becton-Dickinson, or ATTUNE Nxt, Thermo Fisher Scientific, Waltham, MA, USA). For each sample, at least 1 × 10^4^ events in the gate of interest were acquired. Data were analyzed by FCS Express (*DeNovo* software, Los Angeles, CA, USA) and Flowjo (TreeStar, Ashland, OR, USA).

### RNA extraction and quantitative real-time PCR

RNA extraction and quantitative reverse transcriptase–PCR (qRT–PCR) were performed as previously described.^20^ Additional details are reported in the Supplementary Materials and Methods section.

### Gene expression profiling

DCs (3 × 10^6^), obtained from three different HDs, were transfected with either miR-29b or negative control (NC) and co-cultured with U266 MM cells. Twenty-four ours after transfection, cells were collected and separated with immuno-magnetic microbeads. Gene expression profiling was performed as previously described^29^ (additional details are reported in the Supplementary Materials and Methods section). Data set has been deposited under the GEO accession number GSE104831.

Gene Ontology was performed by using DAVID,^30^ whereas analysis of biological pathways modulation by miR-29b was performed by Ingenuity Pathway Analysis (IPA) platform (Ingenuity System, Redwood city, CA, USA).

### Luciferase reporter assay

The 3′-untranslated region of phosphatase and tensin homolog and of its mutant carrying two deletions (100b in length with the center in position 660 and 1728) of the miR-29b target sequence were cloned in pEZX-MT01 vector and purchased from Genecopeia (Rockville, MD, USA). Human embryonic kidney (HEK293) cells were co-transfected with 100 nM of synthetic miR-29b (or miR-NC) and 10 μg of the firefly luciferase reporter vector. Firefly and *Renilla* luciferase activities were measured 48 h after transfection using the Dual-Luciferase assay kit (Promega, Madison, WI, USA) with the Glomax 96 Microplate Luminometer (Promega).

### Western blotting

DCs and MM protein extraction and separation were performed as described elsewhere.^20, 23, 31^ Additional details are reported in the Supplementary Materials and Methods section.

### Immunostaining for confocal microscopy

DCs or MM cells were seeded onto glass coverslips and underwent cytospin for 5 min at 800 r.p.m. Subsequently, cells were washed in PBS, fixed in 4% paraformaldehyde for 12 min, washed three times with PBS, followed by permeabilization with 0.01% Triton-X for 15 min and again washed in PBS containing 0.5% bovine serum abumin. Cells were then incubated with interleukin (IL)-23 antibody (Abcam, Cambridge, UK) or g-H2ax (Cell Signaling, NEB, Hitchin, UK) overnight at 4 °C, washed with PBS three times and incubated with Alexa-flour 488-conjugated secondary antibody (Molecular Probes, Grand Island, NY, USA) for 1 h at room temperature. Cells were again washed three times with PBS and mounted with Vecta-Shield mounting media containing 4',6-diamidino-2-phenylindole. Samples were visualized and images captured using a Leica microscope.

### Cytokines analysis

IL23, CCL2, CXCL10, TNFa, MIP1a, IL10, IL8, VEGFA and IL1b, were detected in supernatant of co-cultures using the BD CBA Human Soluble Protein Flex Set system (Becton Dickinson). Samples were analyzed with a FACSCanto II flow cytometer (Becton Dickinson).

### Migration assay

Chemotaxis was assessed by using 8 μm pore transwell migration assay (Corning Incorporated, Corning, NY, USA). Briefly, 1 × 10^6^ U266 or PBMCs from HDs were washed and resuspended in RPMI1640 medium containing 1% fetal bovine serum. These cells were placed in the upper chamber of the well, whereas the lower chamber contained 50% of supernatant obtained from 29b-DCs/U266 (or 29b-DCs/RPMI8266 or 29b-DCs/MM1S for PBMCs) or NC-DCs/U266 (or NC-DCs/RPMI8226 or MM1S/U266 for PBMCs) 48 h co-cultures. After 5 h (12 h for PBMCs) of incubation at 37 °C 5% CO_2_, cells migrated to the lower chamber were determined by a Trypan-blue count. For PBMCs migration assay, cells migrated in the low chamber were further stained with fluorochrome-conjugated antibodies against CD14, CCR6 and CCR2 (Becton Dickinson), and analyzed with ATTUNE Nxt flow cytometer (Thermo Scientific). Three independent experiments were carried out. Cells from nine different fields were counted for each condition.

### Tube assay formation

Matrigel (50 μl; Corning) were used to coat 96-well plates and allowed to polymerize at 37 °C for 30 min. Human umbilical vein endothelial cells (10^5^) were seeded in each well and then 50 μl of conditioned medium from NC-DCs/29b-DCs + MM cells was added. After 1 h incubation at 37 °C, at least pictures of three representative fields per well were taken using phase contrast microscopy. The tubulogenic potential was quantified by estimating the number of nodes (pixels with at least three neighboring elements corresponding to a bifurcation), segments(elements delimited by two junctions), meshes (areas enclosed by segments or master segments, made by tube-like structures) number and total area, through the ‘Angiogenesis analyzer’ tool (created by Gilles Carpentier, http://image.bio.methods.free.fr/ImageJ/?Angiogenesis-Analyzer-for-ImageJ&lang=en) in ImageJ software (http://imagej.nih.gov/ij/).

### Th17 polarization

Autologous Naive CD4+ T cells were isolated through immunomagnetic sorting by using the human Naive CD4+ T Cell Isolation Kit II (Miltenyi Biotech) and cryopreserved until 2 days before performing the polarization assay, when they were thawed and stimulated with anti-CD3/CD28 microbeads (Miltenyi Biotech). DCs transfected with either NC control (NC-DCs) or synthetic miR-29b (29b-DCs) were prepared as described above. After 48 h co-culture with U266 MM cells, DCs were immunomagnetically separated and co-cultured with autologous Naive CD4+ T cells at a DCs:Lymphocytes ratio of 1:10 for 3 days. Finally, total mRNA was extracted from exposed lymphocytes and the expression of RORC and IL17A (Th17-polarization markers) was assessed through qRT–PCR.

### Th17 expansion

DCs transfected with either NC control (NC-DCs) or synthetic miR-29b (29b-DCs) were loaded with apoptotic (obtained after 24 h treatment with 100 μM Bortezomib^32^) or necrotic (obtained after repeated freeze and thaw cycles^15^) U266 MM cells (1:1 ratio). Tumor-loaded DCs were then used to stimulate autologous CD3+ immunomagnetically separated (Miltenyi Biotech) lymphocytes (DCs-T cells ratio of 1:30). After 5 days of culture, the amount of Th17 cells was quantified through flow cytometry by using fluorochrome-conjugated antibodies against CD4 and CD161.^33, 34^

### Proliferation assay

Modulation of cell proliferation was assessed through Carboxyfluorescein succinimidyl ester staining (Invitrogen) and Cell Counting Kit-8 (CCK-8) assay (Dojindo Molecular Technologies, Mashikimachi, Japan) according to producer guideline. Briefly, 5 × 10^5^ U266 was stained with Carboxyfluorescein succinimidyl ester and co-cultured with either NC or miR-29b transfected DCs (cell ratio 1:1). After 48 h mean fluorescence intensity of MM cells was evaluated through flow cytometry. For CCK-8 assay, 15 × 10^3^ U266 were plated in 96-well plates in the presence of conditioned supernatant from either NC or miR-29b-transfected DCs co-cultured with U266 for 48 h.

### SCID-synth-hu model

The SCID-synth-hu model^35, 36^ was used to evaluate the capability of miR-29b to reduce the production of IL23 in DC-like cells *in vivo*. Additional details are reported in the Supplementary Materials and Methods section.

### Immunohistochemistry

Immunohistochemistry analysis has been performed as described elsewhere.^22^ Additional details are reported in the Supplementary Materials and Methods section.

### Gene expression data sets analysis

Data sets of gene expression profiling of MM or DCs were retrieved from GEO database or from the Multiple Myeloma Research Foundation researcher gateway portal (https://research.themmrf.org). The GSE47552 data set includes data from 5 HDs, 20 patients with monoclonal gammopathy of undetermined significance, 33 high-risk sMM and 41 MM. The GSE40484 data set^17^ includes the gene expression profiling (GEP) profile from inflammatory DCs obtained from cancer-associated ascites (five donors) and normal DCs obtained from four HDs. The CoMMpass (http://research.themmrf.org) (Relating Clinical Outcomes in MM to Personal Assessment of Genetic Profile) Trial (NCT0145429), a longitudinal study in MM relating clinical outcomes to genomic and immunophenotypic include clinical outcomes, Exome-Seq somatic mutations and CN segments, and RNA-Sequencing at pre-treatment of 549 patients at its current release (interim analysis 8).

### Statistical analysis

Differences between means were analyzed by using GraphPad statistical package (GraphPad Software, La Jolla, CA, USA). Parametric and non-parametric tests (always two-sided) were used to compare means between groups, according to Gaussian or not-Gaussian distribution of the variable evaluated. The results were expressed as the mean±s.d. of at least three different experiments. A *P*-value of 0.05 or less was considered statistically significant. Overall survival and progression-free survival (PFS) analyses (Kaplan–Meier curves, log-rank test and Cox regression analysis) have been performed by using SPSS statistical software (IBM Corp, Armonk, NY, USA) on data retrieved by the CoMMpass database.

## Results

### miR-29b is downregulated in tumor-associated DCs

**Figure 1:**
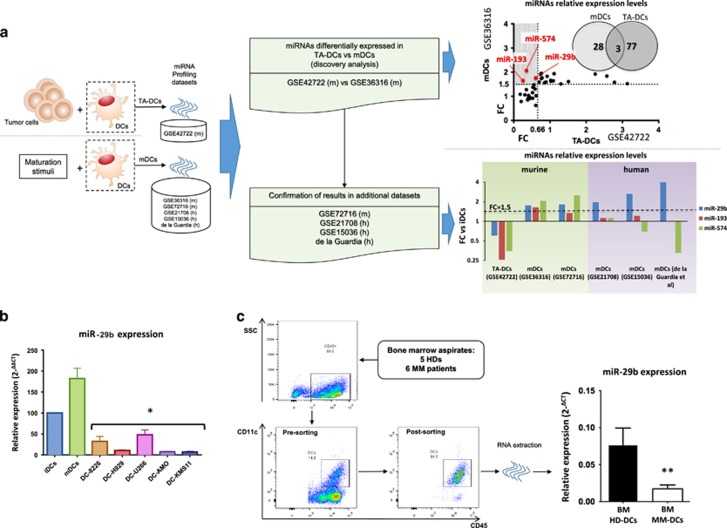
(**a**) Workflow to identify miRNAs differentially expressed in TA-DCs as compared with mDCs. We adopted a two-step approach: in the discovery analysis we compared miRNAs differentially expressed in murine mDCs (data set GSE36316) with murine TA-DCs (data set GSE42722). The shadowed portion of the picture (at the end of the first step) evidences that only three miRNAs are upregulated at least 1.5 times in mDCs and downregulated at least 1.5 times (ratio=0.66) in TA-DCs, among the differentially expressed miRNAs. In the second step we confirmed these results in further four data sets. The results regarding the modulation (with their respective fold changes) of the three miRNAs selected in the ‘discovery’ step in all murine and human data sets evaluated are reported. A line representing the 1.5FC cutoff clearly demonstrated miR-29b as the only miRNA upregulated across all mDCs data sets. (**b**) Relative expression of miR-29b in iDCs, mDCs and DCs co-cultured with five different MM cell lines. All experiments have been repeated at least three times. *P<0.05. (**c**) A representative dot-plot highlighting the gating strategy used to sort DCs from BM aspirates of HDs (BM HD-DCs) or MM patients (MM HD-DCs) and the relative expression of miR-29b in these cells. **P<0.01.

To identify miRNAs differentially expressed between mDCs and TA-DCs as compared with matched iDCs, we analyzed different miRNA microarray data sets (Supplementary Table S1; the flow chart is represented in Figure 1a). The analysis of dataset GSE36316 revealed 28 miRNAs upregulated at least 1.5-fold in murine mDCs as compared with iDCs after *in vitro* stimulation. In a further additional data set, 77 miRNAs were found downregulated in murine DCs after co-culture with either 1D8 or CT-26 tumor cell lines (GSE42722) (Supplementary Table S2). As shown in Figure 1a, comparing both data sets, just three miRNAs, namely miR-574-5p, miR-29b and miR-193, were upregulated in the presence of maturation stimuli (GSE36316), whereas, on the other hand, were downregulated in the presence of cancer cells (GSE42722). We then evaluated the behavior of these selected three miRNAs in a further murine data set GSE72716 and also in three human DC data sets: GSE21708, GSE15036 and a miRNA PCR array performed by Hoces de la Guardia *et al.*^37^ We observed miR-29b as the only miRNA steadily upregulated at least 1.5-fold during DCs maturation across all data sets (Figure 1a). Currently, no data sets exploring miRNA modulation in human DCs co-cultured with cancer cells are available. These *in silico* findings prompted us to investigate miR-29b modulation in human HD-derived DCs after maturation or co-culture with different MM cell lines (RPMI8226, U266, H929, AMO and KMS11). We observed that miR-29b was indeed upregulated during DCs differentiation from monocyte precursors (Supplementary Figure 1A) and after maturation with lipopolysaccharide or co-culture with allogenic lymphocytes (Figure 1b), whereas it was significantly downregulated in DCs after co-culture with all MM cell lines (Figure 1b). To validate our observation in MM patient-derived DCs, we investigated miR-29b expression in CD11c+/CD45+ DCs retrieved from BM of six MM patients and five HDs (Figure 1c) Altogether, DCs represented the 17.67% (±2.67%) of the CD45+/CD138− cells and we were unable to find any difference in term of DCs number or iDCs-mDCs ratio between MM patients and HDs (data not shown). We sorted CD11c/CD45 double-positive cells from BM mononuclear cells and evaluated miR-29b expression levels by qRT–PCR. Consistently with our previous *in vitro* data, we found a significant downregulation of miR-29b in DCs isolated from MM patients as compared with HD (*P*=0.009) (Figure 1c).

These findings suggest that downregulation of miR-29b in DCs exposed to MM cells may be functionally involved in the pathogenesis of MM.

### miR-29b targets multiple genes associated with inflammation

**Figure 2:**
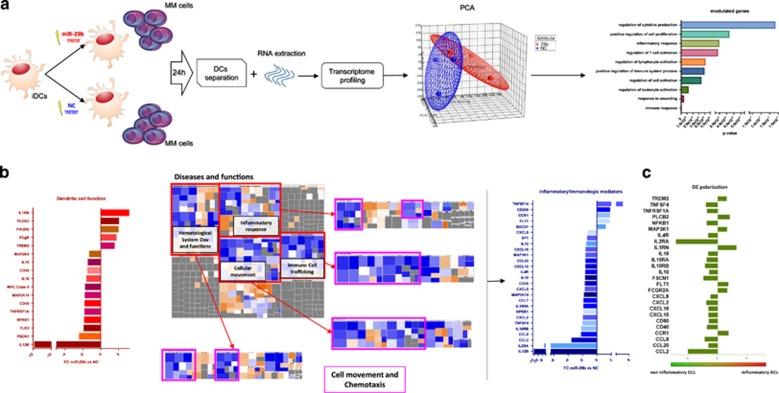
(**a**) Workflow, the results of the principal component analysis (PCA) performed on gene expression data obtained from DCs (from three different donors) transfected with NC or miR-29b and co-cultured with MM cells for 24 h, and the top 10 pathways perturbed by miR-29b enforced expression according to DAVID gene functional annotation tool. (**b**) (i) the magnitude of the modulation of genes belonging to the ‘Dendritic cell function’ canonical pathway affected by miR-29b enforced expression in DCs; (ii) the cellular functions most significantly affected by miR-29b overexpression (purple squares surround cell movement and chemotaxis pathways whose genes are mostly downregulated (blue shift) by miR-29b enforced expression); and (iii) ‘inflammatory/immunologic’ mediators network obtained by merging genes included in those functions and enriched by regulator genes, found to be modulated by miR-29b, with their respective fold changes. All these analyses have been performed through ingenuity pathways analysis software (IPA). (**c**) Anti-inflammatory switch of DCs according to the significant inflammatory/immune genes differentially expressed after miR-29b transfection as compared with inflammatory DCs from GSE40484 data set.

To investigate the potential role of miR-29b in DCs/MM cells cross-talk, we first explored the perturbation induced by enforced expression of miR-29b on DCs transcriptional profile. The experimental design is reported in Figure 2a; briefly, iDCs transfected with miR-29b mimics (29b-DCs) or negative (scramble) control (NC; NC-DCs) were co-cultured with U266 MM cells for 24 h and then analyzed for their transcriptome (transfection efficiency is reported in Supplementary Figure 1B). Differentially expressed genes (DEGs) with at least a 1.5-fold change were considered for further comparative analyses (data deposited under the GEO accession number GSE104831). Unsupervised hierarchical clustering (Supplementary Figure 1C) and principal component analysis (Figure 2a) segregated two subgroups with homogeneous and reproducible transcriptional perturbations induced by miR-29b. A total of 688 genes were found differentially expressed between the two groups. DEGs were then clustered using Gene Ontology analysis (DAVID),^30^ which identified immune-related and inflammatory among the biological functions mainly affected by miR-29b (the top 10 functions and their respective *P*-values are reported in Figure 2b). To understand the biological impact of DEGs, we performed a putative functional investigation by IPA. Among the top 10 perturbed canonical pathways (Supplementary Figure 1D), we chose to investigate the DC maturation signaling, which was the most relevant modulated path in the light of our experimental settings (the pathway is reported in Supplementary Figure 1E). All genes involved in this pathway and modulated by miR-29b are reported in Figure 2c and Supplementary Table S3.

In addition, by evaluating the main functions perturbed in DCs by enforced expression of miR-29b, we found a significant impairment of signaling involved in cell movement and chemotaxis (Figure 2d). We then merged the two main putative networks involved in these cellular functions (Supplementary Figure 1F) to construct a single ‘inflammatory/immunologic mediators’ network. By using IPA tools, we automatically added the IPA-inferred upstream genes to find common regulatory central nodes (the final inferred pathway is showed in Supplementary Figure 2A). Among genes that were convoyed into inflammatory/immunologic mediator pathways, those modulated by miR-29b are reported in Figure 2e and Supplementary Table S3.

Lastly, we hypothesized that perturbation in gene expression produced by enforced miR-29b might polarize DCs; thus, we investigated whether transcriptome changes may be coherent with this phenomenon. To this aim, we took advantage of the ‘comparison analyses’ tool in IPA and we included in the analysis the DEGs comparison from GEP of 29b-DCs versus NC-DCs and the DEGs comparison from the data set GSE40484, matching inflammatory versus normal DCs from peripheral blood.^17^ The final vision that emerged from such comparison clearly indicates that overexpression of miR-29b switches DCs toward a non-inflammatory functional GEP phenotype (Figure 2F and Supplementary Figure 2B). Finally, by qRT–PCR we validated the downregulation of *IL12B, NKFB1, MAP2K4, SP1, CCL2, CXCL8, CCL8, CXCL12, CCL7, CXCL5, IL10, CXCL10 and CXCL16* after enforced expression of miR-29b mimics, underlying the functional polarization of transfected cells (Supplementary Figure 2C).

These data suggest that MM-dependent downregulation of miR-29b promotes a pro-inflammatory GEP phenotype in DCs, providing the rational for functional validation.

### Molecular effects induced in DCs by the enforced expression of miR-29b

To understand the complex regulation exerted by miR-29b on inflammatory/immune pathways, we explored the inferred functional effects of previously identified DEGs in the DC maturation canonical pathway and in the inflammatory/immunologic mediators network by the IPA molecule activity predictor tool. Molecule activity predictor analysis inferred arrest of DCs activation and maturation, based on the following: (i) a predicted reduction in CD83/CD86 expression; (ii) a reduced pro-inflammatory phenotype, due to predicted inactivation of the nuclear factor-κB and signal transducer and activator of transcription 3 (STAT3) signaling; and (iii) an impaired Th1 and, predominantly, Th17 T-lymphocyte polarization (Supplementary Figure 1E). This latter effect is underlined by the finding that *IL12B*, which encodes for IL23 subunit (IL12p40) and *CCL2* (MCP1) were inferred to be deeply downregulated, whereas inactivation of the co-stimulatory molecule CD86 was also predicted. Furthermore, miR-29b overexpression was predicted (and confirmed in qRT–PCR) to modulate a cytokine network involved in angiogenesis and monocyte/neutrophil chemotaxis, which includes IL8 (CXCL8), MCP1, MCP2 (CCL8), MCP3 (CCL7), MIP2a (CXCL2) and IP10 (CXCL10), and finally to abrogate the potent pro-inflammatory STAT3, NFκB and IL8 signaling pathways (Supplementary Figure 2A).

**Figure 3:**
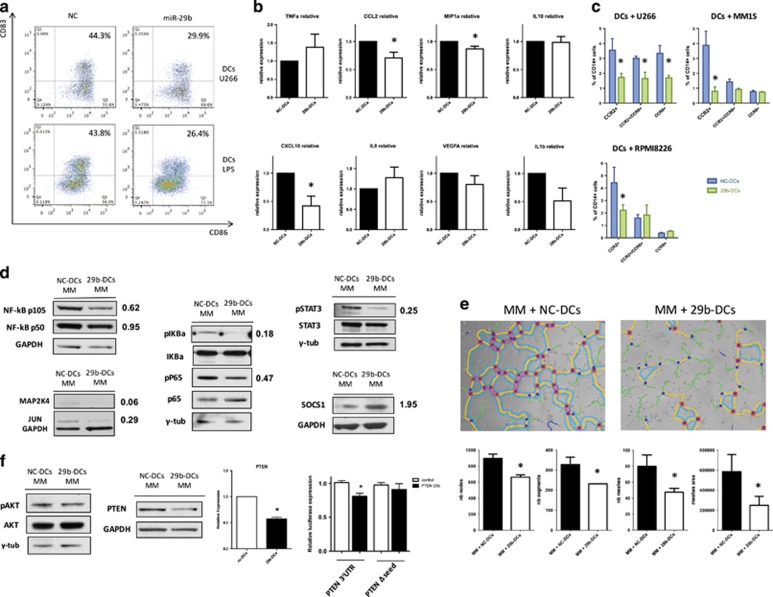
(**a**) Representative flow cytometry analysis of CD86 and CD83 expression on DCs after miR-29b transient transfection and 48 h co-culture with U266 cell lines or with maturation stimuli (lipopolysaccharide). In both cases the percentage of mature DCs is decreased by the enforced expression of miR-29b. (**b**) Evaluation of cytokines production and secretion in the supernatant of DCs after miR-29b or NC transfection and 48 h co-culture with MM cells. Plots represent mean and s.d. of six different experiments. *P<0.05. (**c**) Migration assay to evaluate changes in the capability to attract CCR2+ and/or CCR6+ inflammatory cell populations from PBMCs, between supernatant of 29b-DCs and NC-DCs co-cultured for 48 h with three different MM cell lines (U266, RPMI8226 and MM1S). Plots represent mean and s.d. of three different experiments. *P<0.05. (**d**) Western blot evaluation of the main signaling pathways involved in inflammatory response (NFκB, STAT3, mitogen-activated protein kinase, JUN and suppressor of cytokine signaling 1 (SOCS1)) in DCs after miR-29b transfection and 48 h co-culture with MM cells. (**e**) Results from tubulogenic assay performed in the presence of supernatant from 29b-DCs or NC-DCs co-cultured with RPMI8226 MM cells. Images have been analyzed with ImageJ software and Angiogenesis analyzer plugin. The histograms under the pictures represents the estimation of tubulogenic potential obtained by analyzing the number of nodes (pixels with at least three neighboring elements corresponding to a bifurcation), segments (elements delimited by two junctions), meshes (areas enclosed by segments or master segments, made by tube-like-structures) number and total area. Legend: red points surrounded by blue, nodes surrounded by junctions symbol; red surrounded by yellow, extremities; green, branches (elements constituted by a junction and one extremity); magenta, segments; orange, master segments (segments where none of the two junctions is implicated with one branch); blue sky, meshes; junctions surrounded by red, master junctions (junctions linking at least three master segments); blue and cyan, isolated elements. *P<0.05. (**f**) Evaluation of the AKT signaling (pAKT, AKT and phosphatase and tensin homolog (PTEN)) through western blotting, accompanied by the demonstration of PTEN downregulation at the mRNA level and validation of PTEN as a miR-29b target through luciferase reporter assay. *P<0.05.

To wet-validate all these inferential hypotheses generated through IPA, we next transfected DCs with synthetic miR-29b mimics and evaluated the effects induced by miRNA overexpression in different conditions. Enforced expression of miR-29b mimics in DCs co-cultured with MM cells or lipopolysaccharide significantly reduced double-positive CD83/CD86 mature DCs at 48 h (Figure 3a) and, according to molecule activity predictor prediction, a significant downregulation of the surface expression of the costimulatory molecule CD86 and of the immune regulatory antigen B7H3, which is a validated miR-29 target,^38^ was found (Supplementary Figure 3A). Moreover, a significant decrease in IL-23, CCL2, CXCL10 and MIP1a secretion in the supernatant of 29b-DCs/MM co-cultures (Figure 3b) was detected by flow cytometry microbeads cytokine array. These findings confirmed that miR-29b actually modulates the production and secretion of cytokines by DCs. To investigate the functional relevance of these results, we performed a migration assay where we observed that 29b-DCs co-cultured with U266, MM1S or RPMI8266 disclosed a strongly reduced capability to attract CCR2^+^ pro-inflammatory monocytes from PBMCs, as compared with NC-DCs (Figure 3c). Interestingly, we observed that DCs co-cultured with U266, but not with other MM cell lines, attract a relevant percentage of CCR2+/CCR6+ or CCR6+ monocytes that is reduced by miR-29b transfection in DCs.

Next, we investigated the effects induced by enforced expression of miR-29b on pro-inflammatory molecular networks in DCs after 48 h co-culture with MM cells. According to the *in silico* analysis and mRNA expression data, we observed a reduced expression of both NFKB1 protein subunits p50 and p105 (Figure 3d). In addition, we found reduction of both pIKBa and pP65, indicating downregulation of nuclear factor-κB signaling (Figure 3d). Furthermore, miR-29b antagonized the STAT3 phosphorilation/activation and reduced the expression of MAP2K4 and JUN (Figure 3d and Supplementary Figure 3B). Interestingly, upregulation of the anti-inflammatory protein suppressor of cytokine signaling 1 was detected (Figure 3d). This is consistent with previous findings by our group where transfection of miR-29b-induced suppressor of cytokine signaling 1 promoter demetylation in MM cells.^31^ Moreover, we found a reduced expression of MCL1 (Supplementary Figure 3C), a validated molecular target of miR-29b, whose downregulation has been recently linked with reduced inflammation in lung infections.^39^

Owing to the close interplay between inflammation and angiogenesis, and to the recently disclosed synergistic pro-angiogenetic activity of CCL2 and CXCL16,^40^ we hypothesized that changes in the cytokine/chemokine profile induced by enforced expression of miR-29b in DCs could reduce MM-DC cross-talk-dependent angiogenesis. Indeed, a role of miR-29b in reducing MM-dependent angiogenesis, has been recently demonstrated by our group.^31^ To verify our hypothesis, we cultured early passage human umbilical vein endothelial cells in the presence of conditioned medium obtained from MM (RPMI8226 or U266)/29b-DCs or NC-DCs 48 h co-culture. After 1 h, we observed that NC-DCs conditioned medium induced capillary-like structures to a higher extent as compared with the supernatant derived from 29b-DCs/MM co-culture, as demonstrated by the significantly higher number most indicators of tube formation such as nodes, segments, meshes and meshes area. This latter finding suggest anti-angiogenetic activity for miR-29b in MM-associated BMM (data obtained from supernatant of DCs co-cultured with RPMI8226 are reported in Figure 3e, whereas data obtained from supernatant of DCs co-cultured with U266 are reported in Supplementary Figure 3D).

In addition, due to the important role exerted by inflammasome complex in the DC-dependent inflammatory response and Th17 polarization,^41^ we explored whether enforced expression of miR-29b might perturb also this pathway. We found an overall downregulation of the effector members of this pathway (in particular of Caspase-1) at both messenger and protein levels (Supplementary Figure 3E), a finding that further underlines the anti-inflammatory properties of miR-29b.

To rule out that miR-29b overexpression could produce an overall impairment of DC function, we explored the STAT1 and AKT signaling pathways, which have been reported to have a crucial role for DC survival and activity.^42, 43^ Specifically, we investigated the effects of miR-29b transfection on STAT1 signaling and we found increased STAT1 phosphorylation indicating activation of this pathway (Supplementary Figure 3F). We also evaluated the effects of miR-29b enforced expression on the AKT signaling in DCs. Despite the contrasting effects on this pathway predicted by IPA (Supplementary Figure 3G) and the reported capability of miR-29b to negatively regulate AKT signaling in other systems,^23^ we observed that the phosphorylation of AKT was not affected by miR-29b in DCs. This finding might be at least in part explained by the downregulation of phosphatase and tensin homolog at both protein and mRNA level (Figure 3f). Notably phosphatase and tensin homolog is among the highest predicted target of miR-29b (10 out of 12 databases analyzed by mirDIP, http://ophid.utoronto.ca/mirDIP/) and we validated the targeting specificity by a luciferase reporter assay (Figure 3f).

Altogether, these findings provide hortogonal validation of *in silico* functional inferences, confirming that enforced expression of miR-29b in MM-educated DCs antagonizes their pro-inflammatory activity.

### miR-29b impairs the ability of DCs to generate Th17 lymphocytes

**Figure 4:**
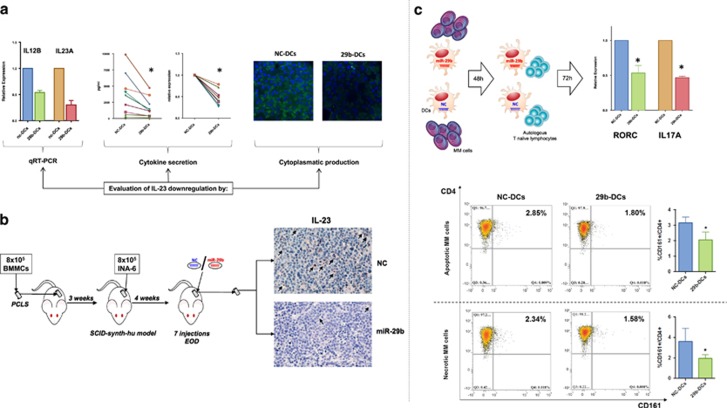
(**a**) Evaluation of the modulation of IL23 in DCs after miR-29b transfection and 48 h co-culture with MM cells, by qRT–PCR, cytokine production and secretion in the supernatant, and intracellular production through confocal microscopy. *P<0.05. (**b**) Overview of the *in vivo* synth-SCID-hu MM model and immunohistochemistry for the detection of DC-like cells (arrows) stained with anti human IL-23. (**c**) In the top part of the picture: workflow and qRT–PCR of RORC and IL17A (the major markers of Th17 polarization) performed on RNA extracted from autologous lymphocytes (naïve Th) after 72 h co-culture with 29b-DCs or NC-DCs previously co-cultured for 48 h with MM cells. *P<0.05. In the bottom part of the picture: representative dot-plots of Th17 (CD4+/CD161+) modulation after miR-29b enforced expression in DCs in the presence or absence of either apoptotic or necrotic U266 MM cell lines. The histograms represent the average of three independent experiments. *P<0.05.

Owing to the important role had by Th17 lymphocytes in pathogenesis of MM,^44^ we investigated whether enforced expression of miR-29b in DCs might result in impaired Th17 polarization, as predicted by *in silico* analysis (see before). In accordance with previous findings, we observed that DCs co-cultured with MM cells were prone to induce Th17 polarization of autologous naive T lymphocytes (Supplementary Figure 4A, B and C). Notably, Th17 polarization is dependent upon IL-1b, IL-6 and IL-23 release by monocytes or DCs.^45^ In this light, GEP analysis showed that *IL12B*, which codes for IL12p40, one of the two sub-units of IL-23 (together with *IL23A* (IL23p19)), was the most downregulated gene after miR-29b transfection. This finding was confirmed by qRT–PCR and a concomitant reduction of IL23A mRNA expression was also observed (Figure 4a). Subsequently, we found that IL-23 was downregulated in the supernatant and in the cytosol of 29b-DCs after 48 h co-culture with U266, as compared with NC-DCs (Figure 4a). Nor *IL6* neither *IL1B* genes were significantly modulated in GEP (data not shown), whereas IL-1b showed a trend to significant reduction at cytokine level (Figure 3b), indicating a pivotal role of IL-23 in mediating miR-29b effects on Th polarization.

To gain translational relevance of previously reported findings, we investigated the effects of miR-29b mimics on IL-23 production *in vivo*. To this end, we used the unique SCID-*synth-hu* model, which recapitulates the huBMM for MM engraftment in a bio-synthetic polymeric scaffold implanted in NOD-SCID mice.^35, 36^ Following huBMM reconstitution, scaffolds were in fact injected with IL-6/BM-dependent MM cells (INA-6) (Figure 4b). SCID-*synth-hu* mice were then treated with miR-29b mimics or NC for 14 days, every other day for a total of 7 injections as previous described.^22^ Scaffolds were retrieved and evaluated by immunohistochemistry for the presence of dendritic-like cells expressing IL-23. We found a significant reduction of dendritic-like cells positive for intracellular expression of human IL-23 (Figure 4b) in the miR-29b treated group, thus providing evidence that miR-29b impairs secretion of this cytokine by DCs *in vivo*. We further evaluated the impact of miR-29b mimics on MM cell proliferation and angiogenesis by staining scaffold-derived tissues for human Ki67 and CD31. As shown in Supplementary Figure 4D, miR-29b antagonized MM cells proliferation (as already reported^22^) and reduced human CD31+ vessels. However, it should be taken into account that these effects represent the result of the whole miR-29b mimics activity on both MM cells and cells of the microenvironment compartment.

Finally, we investigated the functional relevance of impaired IL-23 secretion. We co-cultured for 48 h 29b-DCs and NC-DCs with MM cells. Subsequently, DCs were immune-magnetically purified and co-cultured for 72 h with autologous naïve T lymphocytes (Figure 4c). By qRT–PCR, these lymphocytes were then evaluated for the expression of RORC and IL17A, the major markers of Th17 polarization. We found a significant reduction of both mRNAs in lymphocytes cultured with miR-29b transfected DCs (Figure 4c).

Moreover, it is well known that IL-23 is involved in Th17 expansion and that DCs when loaded with apoptotic or necrotic tumor cells preferentially expand several Th lymphocytes groups.^15^ On this basis, we evaluated whether enforced expression of miR-29b in DCs, loaded with either apoptotic or necrotic MM cells, differentially induces expansion of Th17 cells from autologous CD3+ lymphocytes. After 5 days of lymphocyte–DC co-culture, we observed that transfection of miR-29b mimics led to a significant lower percentage of CD4/CD161 double-positive Th17 cells expansion despite the presence of necrotic or apoptotic MM cells (Figure 4d).

These results led us to conclude that miR-29b antagonizes DCs-mediated Th17 polarization and expansion.

### miR-29b suppression might promote a microenvironmental inflammatory phenotype associated with progression disease and adverse clinical outcome

**Figure 5:**
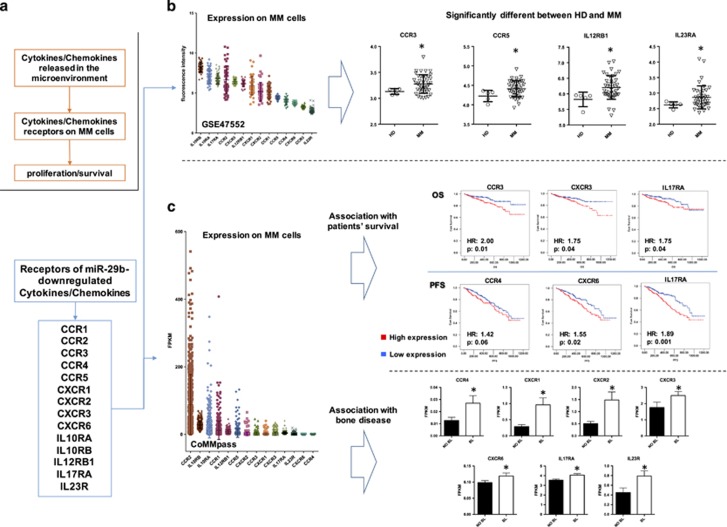
(**a**) Description of the rationale for investigation of expression of the receptors of all cytokines modulated miR-29b in MM cells. (**b**) Evaluation of the expression of the receptors of the main miR-29b modulated chemokines and cytokines on MM cells according to data set GSE47552 (general scatter plots and plots of receptors that significantly differ between HDs (HD) and MM patients). *P<0.05. (**c**) Scatter plots and Kaplan–Meier curves with log-rank test results of the chemokine/cytokine receptors able to significantly discriminate PFS, overall survival (OS) and patients presenting bone disease (BL) as compared with patients that do not present lytic lesions (No BL) in the coMMpass trial. For survival analysis, patients were grouped into high and low expression groups according to the median value of receptor expression (FPKM). *P<0.05.

Based on our finding demonstrating that transfection of miR-29b mimics significantly downregulates directly or indirectly a variety of inflammatory cytokines and chemokines at mRNA and/or protein level, we investigated the putative role of the known receptors of these molecules (*CCR1, CCR2, CCR3, CCR4, CCR5, CXCR1, CXCR2, CXCR3, CXCR6, IL10RA, IL10RB, IL12RB1, IL17RA and IL23R*) in MM disease pathobiology by performing an *in silico* analysis on MM gene expression data sets. Indeed, our working hypothesis was that these cytokines, secreted within huBMM mainly by DCs, could elicit a pro-tumor activity, which might be counteracted by miR-29b (Figure 5a).

We evaluated the differential expression of these receptors between tumor and normal PCs in the GSE47552 data set. This data set includes gene expression profiling of CD138+ purified PCs from HDs, monoclonal gammopathy of undetermined significance, smoldering MM and MM patients. During disease evolution, several of these genes demonstrated a progressive downregulation (*CCR2, IL10RA and IL10RB*), some a progressive upregulation (*IL12RB1 and IL23RA*) and others became upregulated only in certain phases of the disease (Supplementary Figure 5A). Among others, we found significant upregulation of *CCR3, CCR5* and both components of IL-23 receptor (*IL12RB1 and IL23RA*) in patient-derived MM cells (Figure 5b), whereas both components of the IL-10 receptor were significantly downregulated, thus suggesting a pro-inflammatory unbalancing of the MM *niche*. According to previous reports,^46^ we confirmed a significant downregulation of *CCR2* (Supplementary Figure 5A).

Then, we evaluated the association of the expression of these receptors with patients’ outcome taking advantage of data from CoMMpass Trial (NCT0145429), a longitudinal study in MM, relating clinical outcomes to genomic and immune-phenotypic profiles of CD138^+^ selected PCs from the BM of newly diagnosed MM patients. In its currently available release (interim analysis 8), RNA-Sequencing, together with clinical data, was available for 549 MM patients. Patients were divided into two subgroups according to the median expression of each of the investigated chemokine/cytokine receptors. We observed that: (i) patients with high expression of *CCR3 and CXCR3* experienced a significantly worse outcome, (ii) patients with high expression of *CCR4 and CXCR6* experienced a shortest PFS after frontline treatment and (iii) patients with high expression of *IL17RA* presented a significantly shorter overall survival and PFS (Figure 5c). All other receptors were not associated with any changes in survival outcomes. We then performed a multivariate Cox regression analysis to evaluate the independent prognostic role of all these genes within recognized prognostic scores of MM, such as international staging system (ISS) and its revised version. Importantly, revised-ISS combines ISS with chromosomal abnormalities and lactate dehydrogenase levels, improving MM patients’ stratification into more homogeneous subgroups.^47^ As reported in Supplementary Figure 5B, we confirmed the independent prognostic relevance of *IL17RA* (hazard ratio of 1.82 and 1.95 for PFS in the presence of ISS or revised-ISS, respectively; hazard ratio of 1.84 and 2.26 for overall survival in the presence of ISS or revised-ISS, respectively), of *CCR3* in overall survival and of *CXCR6* in PFS (Supplementary Figure 5B).

Taking into account the role had by inflammation in the pathogenesis of MM-related bone disease,^13, 44^ we explored in the CoMMpass database, the expression of the known receptors of miR-29b-downregulated cytokines/chemokines in MM patients with bone lytic lesions, as compared with MM patients without bone disease. We found that PCs from MM patients with bone lesions presented a significantly higher expression of *CCR4, CXCR1, CXCR2, CXCR3, CXCR6, IL17RA and IL23R* (Figure 5c).

Altogether, these data underscore the relevance of an inflammatory microenvironment in the progression, outcome and pathogenesis of MM, and the potential role of miR-29b in antagonizing these mechanisms.

### Enforced expression of miR-29b in DCs antagonizes survival signaling in MM cells

**Figure 6:**
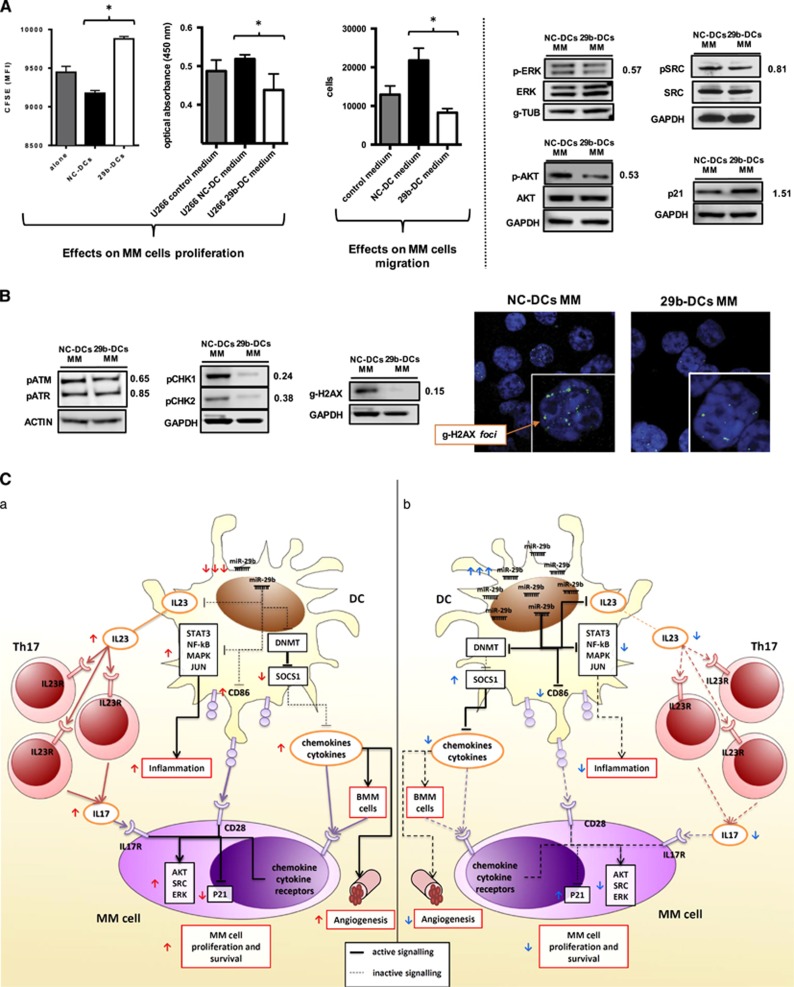
(**A**) The left and center histograms report the results of the proliferation assays performed on MM cells (U266) stained with Carboxyfluorescein succinimidyl ester and co-cultured with NC-DCs or 29b-DCs, or cultured in the presence of conditioned medium obtained from MM/NC-DCs and MM/29b-DCs co-cultures, respectively. The right histogram represents the number of MM cells attracted by the conditioned medium obtained from MM/NC-DCs and MM/29b-DCs co-cultures (migration assay). The blots on the right side of the panel represent the main survival and proliferation signaling (ERK, AKT, SRC and p21) in MM cells evaluated after 48 h co-culture with NC-DCs or 29b-DCs by western blotting. *P<0.05. (**B**) Left, evaluation of DNA damage response activation in MM cells (U266) co-cultured with either NC-DCs or 29b-DCs in western blotting; right, evaluation of g-H2AX nuclear foci (DNA double-strand break markers) in confocal microscopy in MM cells (U266) co-cultured with either NC-DCs or 29b-DCs. (**C**) This cartoon shows the main molecular and functional changes induced by miR-29b enforced expression in DCs in the context of MM microenvironment. (**a**) The ‘normal’ pathologic status, in which MM cells induce a downregulation of miR-29b in DCs thus promoting an inflammatory microenvironment that leads to a survival advantage. (**b**) The changes that we demonstrated occuring after miR-29b transfection in DCs.

On the basis of above *in silico* findings demonstrating that MM cells express inflammatory cytokine receptors, which may be associated with worse outcome, and taking into account that DCs have been described as pro-survival components of the MM-associated BM niche,^4, 11, 12^ we hypothesized that enforced expression of miR-29b in DCs might antagonize the growth of co-cultured MM cells. Accordingly, we evaluated in proliferation assays whether miR-29b-transfected cells, or the supernatant obtained by a 48 h co-culture of 29b-DCs or NC-DCs with U266, might affect MM cells proliferation and migration. We found that transfection of miR-29b in DCs significantly reduced their ability to support both growth and motility of MM cells (Figure 6A). This event appears to be related to reduced phosphorylation of ERK, AKT and, to a lesser extent, SRC in MM cells (Figure 6A). Furthermore, we observed a consistent increase in p21, suggestive of slower cell cycle progression (Figure 6A), together with a slight upregulation of cPARP (Supplementary Figure 5C), whereas BCL2 expression remained unchanged (Supplementary Figure 5C).

DCs and the inflammatory huBMM have been also reported to induce genomic instability in MM cells, an event that under specific conditions could promote the arising of mutations responsible for tumor progression, drug-resistance and immune escape.^48^ Indeed, the interaction between DCs and MM cells led to the induction of DNA double-strand breaks that are significantly reduced by enforced expression of miR-29b in DCs. Specifically, we found that MM cells co-cultured with 29b-DCs presented a reduction of the phosphorylation of ATM, ATR, of their downstream molecules CHK1 and CHK2 and of H2AX, the main double-strand break marker, in both protein and foci numbers (Figure 6B) as compared with MM cells co-cultured with NC-DCs.

Overall, these findings provide evidence of a tumor promoting MM/DCs cross-talk, which is specifically antagonized by enforced expression of miR-29b in DCs.

## Discussion

DCs are relevant components of MM-associated BM niche and support growth, proliferation and drug-resistance of MM cells.^4, 11, 13^ Based on recent experimental evidence of a pivotal role of miRNAs in regulating DCs function,^27^ we hypothesized that MM sustaining activity might be dependent on DCs reprogramming at miRNA level. To experimentally address this hypothesis, we first investigated by *in silico* analysis miRNAs predicted as differentially expressed in TA-DCs and mDCs, and thereafter, a wet validation was performed in DCs cultured with MM cells. Among differentially expressed miRNAs, we identified miR-29b as the only miRNA downregulated in TA-DCs, whereas upregulated in physiologic activation of DCs. We confirmed this finding in MM-associated DCs either from DC/MM-cells co-cultures or directly from BM of MM patients. To address the mechanism of miR-29b activity in MM-educated DCs, we enforced miR-29b expression in DCs cultured with MM cells and then we performed a GEP analysis. MiR-29b antagonized polarization of DCs to a pro-inflammatory phenotype and this effect mainly relied on the impairment of DCs pro-inflammatory machinery at different levels, including inhibition of nuclear factor-κB, STAT3, mitogen-activated protein kinase and JUN activity (Figure 6C and Table 1). A crucial finding was the decrease of IL-23, confirmed *in vivo*, and of other pro-inflammatory cytokines/chemokines that in turn impaired attraction of inflammatory cells, polarization and expansion of Th17 lymphocytes. These observations are in line with a recent report in Crohn’s disease,^16^ where overexpression of miR-29a (which shares the same seed sequence of miR-29b) in DCs reduced their capability to polarize Th17 cells and ameliorated the symptoms in a mouse model of inflammatory colitis. We think that our results may be of specific interest taking into account that immune cell infiltration and chronic inflammation have been widely associated with cancer development, progression and patients’ prognosis.^49, 50, 51, 52^ In addition, different cytokines involved in inflammation, including IL-23, TNF-a, IL-6, IL-10, IL-1b and IL-17, are highly upregulated in MM patients’ BM and actively participate to MM and bone disease pathogenesis.^2, 44, 53^ Specifically, there is emerging evidence of increased frequency of pro-inflammatory Th17 lymphocytes in the microenvironment of different cancers, including MM.^15, 53, 54^ These cells are induced to differentiate from naïve T cells by inflammatory DCs in the presence of IL-23, IL-1b and IL-6, and represent the main source of IL-17,^17^ a recently validated growth factor for MM and a potent inducer of osteoclast activity.^3, 44, 53^ Moreover, Th17 cells may be attracted in the BM by different chemokines including CCL2.^55, 56^ Considering these findings, our results indicate that miR-29b impairs the recruitment and polarization of Th17 by reducing IL-23 and CCL2 secretion, thus abrogating the tumor-sustaining activity of inflammatory MM-associated huBMM. The latter effect is further supported by the capability of miR-29b to antagonize DCs pro-angiogenetic potential, as here demonstrated. Lastly, an inflammatory microenvironment has been further related to susceptibility to DNA damage and, in turn, to an increased risk of cancer initiation and progression.^57, 58^ Indeed, different authors have reported the capability of MM-educated DCs to induce MM cells proliferation, chemo-resistance and genomic instability.^4, 12, 48^ All together, these findings highlight the importance of huBMM-derived stimuli on MM survival and progression, and points the DC-MM cells interaction and inflammation as potential targets for anti-MM approaches. At this aim, we indeed demonstrated that transfection of miR-29b in DCs efficiently impairs pro-survival signaling in MM. This is highlighted by downregulation of ERK, AKT and SRC activity, and upregulation of p21 in MM cultured with miR-29b-transfected DCs. Interestingly, 29b-DCs induced less double-strand breaks in MM cells, as proven by the decrease in g-H2AX and the reduction of DNA-damage signaling activation. In this view, it is possible to speculate that the capability of miR-29b to reprogram DCs to exhibit an anti-inflammatory phenotype disrupts the DCs–MM cells axis and induces significant molecular changes in MM cells. To our knowledge, this is the first report where changes in DCs polarization by miRNA overexpression produce relevant changes in co-cultured MM cells, providing a formal proof that a tumor promoting MM/DCs cross-talk indeed occurs within the protecting BMM, which is specifically antagonized by miR-29b. Along the same line, by exploring two tumor gene expression data sets from MM patients, we provided evidence that an inflammatory huBMM sustains MM cell growth and development of bone disease in patients and affect their outcome. Indeed, we found that MM patients, whose PCs overexpress different cytokine and chemokine receptors including IL23R and IL17RA, present a worse outcome and are more likely to experience bone lytic lesions. This finding is relevant considering that DCs are among the main source of ligands for these receptors and that enforced expression of miR-29b in DCs indeed reduces their production and secretion. In addition, these results further underscore the relevance of the Th17 response in the pathogenesis of MM.

Altogether, these findings are of clinical interest, taking into account recent clinical trials, where anti-inflammatory agents such as aspirin and curcumin revealed a promising therapeutic activity in both monoclonal gammopathy of undetermined significance and sMM patients.^59, 60^ Moreover, the actual role of miR-29b within DCs in the different stages of disease evolution, from monoclonal gammopathy of undetermined significance to overt MM, will be investigated in follow-up studies.

In the general view of miR-29b anti-inflammatory potential, we already reported that its overexpression in osteoclast precursors reduced their bone lytic capability;^25^ In addition, we observed that enforced expression of miR-29b in MM cells could affect survival and angiogenesis through suppressor of cytokine signaling 1 upregulation, and VEGFA and IL8 downregulation.^22, 23, 31, 61^ Here we show that miR-29b overexpression in DCs affect their pro-inflammatory and pro-tumor potential through different molecular mechanisms. Taking into account the prominent role had by chronic inflammation in MM pathology, these results indicate that miR-29b deregulation in different cell components of the BMM as well as in MM cells has a major role in disease development and progression, directly modulating BMM and inducing bone destruction. Our findings provide therefore the rationale for the development of a miR-29b-based treatment for MM.

## Figures and Tables

**Table 1:** Main effects induced by miR-29b enforced expression in DCs

*Affected function*	*Effects*	*Traslational relevance*
DC maturation	↓ CD83/86 double-positive mature DCs	
Production and secretion of cytokines and chemokines	↓ IL-23, CCL2, CXCL10, IL1β, MIP1α IL8, CCL8, CCL7, CXCL2	
Chemotaxis	↓ Capability of DCs to attract CCR2+ pro-inflammatory monocytes	Impairment and recover of the inflammatory-immunosuppressive human BM milieu, which strongly contribute to MM progression, bone disease and immune escape
Th17 polarization and expansion	↓ IL-23 ↓ RORc, IL-17A ↓ Th17	
Pro-inflammatory molecular networks	↓ NF-κB, STAT3, MAP2K4 ↑ SOCS1	
Inflammasome machinery	↓ Caspase1, BIRC3	
Angiogenesis	↓ Ability to develop tube-like structures	Reduction of MM cell growth and extramedullary dissemination
Survival signaling in MM co-cultured with DCs	↓ ERK, AKT, SRC ↑ P-21, c-PARP	Decrease in MM cells proliferation
Genetic instability in MM co-cultured with DCs	↓ p ATM, pATR, CHK1, CHK2, H2AX	Reduction of inflammation-related DNA damage and potentially of mutations responsible for tumor progression, drug resistance and immune escape

Abbreviations: BM, bone marrow; DC, dendritic cell; IL-23, interleukin-23; MM, multiple myeloma; NF-κB, nuclear factor-κB; SOCS1, suppressor of cytokine signaling 1; STAT3, signal transducer and activator of transcription 3.
