# Supplementary material for: MiR-29b antagonizes the pro-inflammatory tumor-promoting activity of multiple myeloma-educated dendritic cells
Source: Leukemia. 2018 Jan 30;32(4):1003–15. doi: 10.1038/leu.2017.336 (PMC5886056; doi:10.1038/leu.2017.336)

Supplementary Material and methods

DCs generation

CD14+ cells were cultured for 6 days in RPMI-1640 medium containing 10% heat inactivated fetal bovine serum, 1% penicillin/streptomycin, 100 ng/ml recombinant human GM-CSF (Milteny Biotech, Gladbach, Germany) and 50 ng/ml IL-4 (Milteny Biotech, Gladbach, Germany) at 37°C in 5% CO2 humidified atmosphere. Half-medium was replaced every 48 hours. To estimate immature DCs enrichment, the culture underwent flow-cytometry for the expression of CD14, CD11c, HLA-DR, CD86, CD83. Physiologic DC maturation was reached by coculture with healthy donor allogenic lymphocytes or by exposure to LPS (50 ng/ml) as maturation stimuli. the expression of maturation markers was assessed by flow cytometry.

RNA extraction and quantitative real-time-PCR

Total RNA from DCs was extracted by using miRVANA PARIS (Invitrogen), Taq-Man® MicroRNA assays (Life Technologies) was used to detect and quantify mature mir-29b, according to manufacturer's guidelines on a ViiA7 System (Life Technologies). MiR-29b expression was normalized on RNU44 (Life Technologies). cDNA for single gene expression was obtained using the high capacity cDNA reverse transcription kit (Life Technologies) and then used to quantify IL12B, IL23A, IL17A, RORC, FOXP3, MAP2K4, NFKB1, SP1, PTEN, CCL2, CXCL8, CCL8, CXCL12, CCL7, CXCL5, IL10, CXCL10 and CXCL16; GAPDH was used for normalization. Both miRNA and mRNA expressions were quantified using the $2^{-\Delta\Delta CT}$ method and expressed as the relative fold change of target miRNA/mRNA normalized to the RNU44/GAPDH housekeeping gene.

Gene-expression Profiling

Total RNA (tRNA) from DCs was extracted through column purification with RNeasy kit (Qiagen, Hilden, Germany). A total of 300 ng RNA were used as starting material for preparing the hybridization target by using the Ambion® WT Expression Kit (Ambion, Life Techologies). The integrity, quality and quantity of tRNA were assessed by the Agilent Bioanalyzer 2100 (Agilent Technologies, Santa Clara, CA) and NanoDrop 1000 Spectrophotometer (Thermo Scientific, Wilmington, DE). The

amplification of cRNA, the clean up and the fragmentation were performed according to the Affymetrix's procedures. Microarray data were generated by Human transcriptom array 2.0 ST (Affymetrix Inc., Santa Clara, Ca) containing over 6 million distinct probes targeting coding transcripts, exon-exon splice junction and non-coding transcript. Arrays were scanned with an Affymetrix GeneChip Scanner 3000. Raw data produced by the Affymetrix Platform (i.e. CEL files) were processed and RMA normalized using Affymetrix Expression Console (EC). Clustering and fold-change analysis were done by using transcription array console (TAC, Affymetrix).

Western blot

DCs and MM cells were lysed in NP40 CellLysis Buffer (Novex) containing a cocktail of protease and phosphatase inhibitors (Thermo Scientific, Waltham, MA). Whole cells lysates (20-30 ug/well) were loaded and separated on 4–12% NovexBis–Tris SDS–acrylamide gels (Gibco, Life Technologies). Proteins were then transferred on nitrocellulose membranes by Trans-Blot Turbo Transfer Starter System (Bio-Rad, Berkeley, CA). After protein transfer the membranes were blotted with the following primary antibodies: anti-STAT3 (Cell Signaling, #9131), anti-pSTAT3 (Cell Signaling, #9145), anti-SOCS1 (Santa Cruz biotechnology, 9021), anti-AKT (Cell Signaling, #9272), anti-pAKT (Cell Signaling, #4060), anti-pSTAT1 (Cell Signaling, #8826), anti-STAT1 (Cell Signaling, #9175), anti-p21 (Cell Signaling, #2946), anti-pERK (Cell Signaling, #4370), anti-ERK (Cell Signaling, #9107), anti-pIKBa (Cell Signaling, #2859), anti-IKBa (Cell Signaling, #4814), anti-gammaH2X (Cell Signaling, #9718), anti-pATM (Cell Signaling, #5883), anti-pATR (Cell Signaling, #2853), anti-pCHEK1 (Cell Signaling, #2348), anti-pCHEK2 (Cell Signaling, #2197), anti-pSRC (Cell Signaling, #2105), anti-SRC (Cell Signaling, #2123), anti-MCL1 (Cell Signaling, #5453), anti-cMYC (Cell Signaling, #5605), anti-NF-kB1p105/p50 (Cell Signaling, #12540), anti-pP65 (Abcam, 76302), anti-P65 (Abcam, 32536), (Santa Cruz Biotechnology, 56735), anti-MAP2K4 (Santa Cruz Biotechnology, 964), anti-JUN (Santa Cruz Biotechnology, 1694), anti-PARP (Cell Signaling #9532), anti-BCL2 (Santa Cruz Biotecnology, 7382), anti-PTEN (Cell Signaling, #9188), anti-gamma-tubulin (Santa Cruz Biotechnology, 7396), GAPDH (Santa Cruz Biotechnology, 25778), anti-B7-H3 (Abcam, 134161).Subsequently, blots were incubated with goat anti-mouse or goat anti-rabbit antibodies HRP-conjugated (Santa Cruz

Biotechnology). Immunoreactive bands were detected by use of enhanced chemiluminescence (ECL) method, acquired through the C-DIGIT scanner (LI-COR) and quantified by Image Studio Lite 5.0 (LI-COR).

SCID-synth-hu model

In detail, a three-dimensional (3D) bone-like poly-Σ-caprolactone polymeric scaffold (PCLS), presenting interconnected large (100–300 mm) and small pores (1–10 mm) resembling the micro-architecture of a normal human adult bone, was implanted into a 6-weeks old female SCID mouse and seeding of BMMCs into PCLSs was performed. Briefly, A suspension of 8×105 cells in 500 μl of growth medium was threaded into two ending faces of the cylindrical scaffold. Before surgical implantation into a SCID mouse flank, PCLSs were incubated in complete medium at 37°C in 5% CO2 for 24 h to allow cell adhesion on 3D surfaces. Chloralium hydrate anesthesia (400 mg/kg, 0,15 ml) was used during all surgical procedures. After three weeks, 8×105 BM-dependent INA-6 MM cells were injected in vivo into previously implanted PCLSs. Approximately one month later, when sIL6R became detectable in mice sera, miR-29b or negative control (NC) were injected directly into the scaffold (total of 7 injections, 2 days apart). Neutral lipid emulsion (NLE) (MaxSuppressor in vivo RNA Lancer II, BIOO Scientific, Austin, TX) was used for the administration of synthetic miR-29b or NC, according to the manifacturer's instructions.

In vivo effects induced by miR-29b were then evaluated by immunohistochemistry on retrieved scaffolds at the end of treatments.

Immunohistochemistry

For each case 4 μm-thick serial sections were cut from a representative block of formalin fixed, paraffin-embedded tissue, mounted on acid-cleaned glass slides, and heated at 55 °C for 60 minutes. Slides were, dewaxed with xylene, and processed for hematoxylin and eosin and immunohistochemistry. All the procedures were performed at room temperature. Immunohistochemical evaluation of anti-IL-23/Ki67/CD31 was performed using the LSAB+ System HRP (DakoEnvision System, CA), followed by the addition of 3,3′-diaminobenzidine as a chromogen. Endogenous peroxidase activity

was quenched for 5 minutes in 3% hydrogen peroxidase, and the slides were rinsed in wash solution (TBST, 0.05 mol/l Tris Buffered Saline with Tween20). Antigen retrieval was performed with EDTA buffer pH 9 for 30 minutes at 98 °C. Slides were washed three times in phosphate-buffered saline (PBS; pH 7.4) for 5 minutes. Immunostaining was performed using a purified mouse monoclonal antibody anti-human IL-23 (Abcam, 1:150 dilution), anti-Ki67 (Dako, 1:150 dilution) and anti-human CD31 (Dako, 1:40 dilution) for 1 hour at 25 °C. Sections were finally counterstained with hematoxylin. Negative controls were performed in each run by substituting primary antibodies with antibodies with irrelevant specificity but of the same isotype of the primary antibodies.

Data availability

All the data supporting the findings and results of this work are available upon request from the corresponding author.

Supplementary Table 1

Database	GEO	Platform	Institute	Organism	Summary
**1**	GSE36316	miRCURY LNA microRNA Array (GPL7722)	Seattle Biomedical Research Institute	Mouse	miRNA expression profiling of primary murine splenic dendritic cells (Flt3L expanded) comparing untreated cells to cells infected with Influenza A or stimulated with polyI:C in vitro.
**2**	GSE42722	miRCURY LNA™ microRNA Array (GPL16352)	Nankai University	Mouse	miRNA expression profiling in tumor-associated BMDCs induced by tumor cells 1D8 or CT-26 in comparison with those cultured in medium.
**3**	GSE72716	Agilent-019119 Mouse miRNA Microarray 1.0 (GPL8824)	Oregon Health & Science University	Mouse	miRNA expression profile of immature and CpG stimulated murine BMDCs.
**4**	GSE21708	Multi-species 1.1K miRNA array (GPL9517)	University of Minnesota	Human	miRNA expression profiling of human monocyte, immature DCs (imDCs) and mature DCs(mDCs).
**5**	GSE15036	Agilent-019118 Human miRNA Microarray 2.0	Kunming institute of zoology.CAS	Human	miRNA expression profiling of human monocyte, immature DCs (imDCs) and mature DCs(mDCs).
**6**	**N/A** (source: de la Guardia AH et al.)	Human Immunopathology miRNA PCR Assay (Qiagen, 88 miRNAs)	University of Bordeaux	Human	miRNA expression profile of immature and *H. Pylori* stimulated human monocyte-derived DCs.

Supplementary table 2

mDCs vs iDCs >1.5 uFC	TA-DCs vs iDCs >1.5 dFC
hsa-miR-296-5p/mmu-miR-296-5p/rno-miR-296*	mmu-miR-29b
mmu-miR-155	mmu-miR-96
mmu-miR-483*/rno-miR-483	mmu-miR-299*
hsa-miR-574-5p/mmu-miR-574-5p	mmu-miR-138*
mmu-miR-466a-5p	mmu-miR-705
mmu-miR-881*	mmu-miR-742
hsa-miR-223/mmu-miR-223/rno-miR-223	mmu-miR-291a-3p
mmu-miR-466b-5p	mmu-miR-343
mmu-miR-669c	mmu-miR-192
mmu-miR-466e-5p	mmu-miR-27a*
mmu-miR-712*	mmu-miR-125a-3p
hsa-miR-142-3p/mmu-miR-142-3p/rno-miR-142-3p	mmu-miR-193b
mmu-miR-466d-5p	mmu-miR-421
hsa-miR-29b/mmu-miR-29b/rno-miR-29b	mmu-miR-551b
mmu-miR-466c-5p	mmu-miR-31
hsa-miR-494/mmu-miR-494/rno-miR-494	mmu-miR-124
hsa-miR-193a-3p/mmu-miR-193/rno-miR-193	mmu-miR-208a
mmu-miR-468	mmu-miR-878-5p
mmu-miR-466f-5p/mmu-miR-466f	mmu-miR-29b*
hsa-miR-101/mmu-miR-101a/rno-miR-101a	mmu-miR-532-3p
hsa-miR-297/mmu-miR-297a	mmu-miR-29c
mmu-miR-297c	mmu-miR-673-3p
hsa-miR-33a/mmu-miR-33/rno-miR-33	mmu-miR-181b
mmu-miR-669a	mmu-miR-719
hsa-miR-206/mmu-miR-206/rno-miR-206	mmu-miR-759
hsa-miR-142-5p/mmu-miR-142-5p/rno-miR-142-5p	mmu-miR-694
mmu-miR-715	mmu-miR-693-5p
hsa-miR-22*/mmu-miR-22*/rno-miR-22*	mmu-miR-216b
	mmu-miR-433
	mmu-miR-296-3p
	mmu-miR-758
	mmu-miR-293
	mmu-miR-380-5p
	mmu-miR-200c
	mmu-miR-128
	mmu-miR-717
	mmu-miR-203*
	mmu-miR-701
	mmu-miR-374
	mmu-miR-411
	mmu-miR-23b
	mmu-miR-92b
	mmu-miR-202-3p
	mmu-miR-338-5p
	mmu-miR-708
	mmu-miR-143
	mmu-miR-190b

mmu-miR-433*
mmu-miR-138
mmu-miR-654-5p
mmu-miR-124*
mmu-miR-201
mmu-miR-654-3p
mmu-let-7c-1*
mmu-miR-708*
mmu-miR-181d
mmu-miR-93*
mmu-miR-345-3p
mmu-miR-486
mmu-miR-193
mmu-miR-218
mmu-miR-24-2*
mmu-miR-764-5p
mmu-miR-449b
mmu-miR-149
mmu-miR-26b*
mmu-miR-27b*
mmu-miR-28*
mmu-let-7g*
mmu-miR-875-5p
mmu-miR-574-5p
mmu-miR-30b
mmu-miR-224
mmu-miR-148a*
mmu-miR-370
mmu-miR-127

Supplementary table 3. Main genes and networks modulated by miR-29b in DCs

GENE SYMBOL	FULL NAME (ENTREZ GENE NAME)	LOCATION	FOLD CHANGE
**DENDRITIC CELL FUNCTION**			
**IL1RN**	interleukin 1 receptor antagonist	Extracellular Space	3.13
**PLCB2**	phospholipase C beta 2	Cytoplasm	2
**PIK3R6**	phosphoinositide-3-kinase regulatory subunit 6	Cytoplasm	1.93
**FCGR1B**	Fc fragment of IgG receptor Ib	Plasma Membrane	1.83
**FCGR1A**	Fc fragment of IgG receptor Ia	Plasma Membrane	1.76
**FCGR2A**	Fc fragment of IgG receptor IIa	Plasma Membrane	1.58
**TREM2**	triggering receptor expressed on myeloid cells 2	Plasma Membrane	1.5
**MAP2K4**	mitogen-activated protein kinase kinase 4	Cytoplasm	-1.23
**IL10**	interleukin 10	Extracellular Space	-1.4
**CD80**	CD80 molecule	Plasma Membrane	-1.46
**HLA-DOA**	major histocompatibility complex, class II, DO alpha	Plasma Membrane	-1.53
**HLA-DQB1**	major histocompatibility complex, class II, DQ beta 1	Plasma Membrane	-1.56
**IL18**	interleukin 18	Extracellular Space	-1.57
**HLA-DMA**	major histocompatibility complex, class II, DM alpha	Plasma Membrane	-1.69
**MAP3K14**	mitogen-activated protein kinase kinase kinase 14	Cytoplasm	-1.74
**CD40**	CD40 molecule	Plasma Membrane	-1.76
**TNFRSF1A**	tumor necrosis factor receptor superfamily member 1A	Plasma Membrane	-1.77
**NFKB1**	nuclear factor of kappa light polypeptide gene enhancer in B-cells 1	Nucleus	-1.82
**TLR3**	toll-like receptor 3	Plasma Membrane	-1.87
**HLA-DMB**	major histocompatibility complex, class II, DM beta	Plasma Membrane	-1.89
**HLA-DQA1**	major histocompatibility complex, class II, DQ alpha 1	Plasma Membrane	-1.91
**FSCN1**	fascin actin-bundling protein 1	Cytoplasm	-2.39

**IL12B**	interleukin 12B	Extracellular Space	-11.81
**INFLAMMATORY/IMMUNOLOGIC MEDIATORS**			
**TNFSF14**	tumor necrosis factor superfamily member 14	Extracellular Space	5.48
**CD209**	CD209 molecule	Plasma Membrane	1.79
**CCR1**	chemokine (C-C motif) receptor 1	Plasma Membrane	1.77
**FLT1**	fms related tyrosine kinase 1	Plasma Membrane	1.76
**SOCS1**	suppressor of cytokine signaling 1	Cytoplasm	1.16
**CXCL8**	chemokine (C-X-C motif) ligand 8	Extracellular Space	-1.22
**SP1**	Sp1 transcription factor	Nucleus	-1.29
**IL10**	interleukin 10	Extracellular Space	-1.4
**CXCL16**	chemokine (C-X-C motif) ligand 16	Extracellular Space	-1.51
**CCL20**	chemokine (C-C motif) ligand 20	Extracellular Space	-1.53
**MAP3K1**	mitogen-activated protein kinase kinase kinase 1, E3 ubiquitin protein ligase	Cytoplasm	-1.53
**CXCL10**	chemokine (C-X-C motif) ligand 10	Extracellular Space	-1.54
**IL4R**	interleukin 4 receptor	Plasma Membrane	-1.55
**IL18**	interleukin 18	Extracellular Space	-1.57
**CD36**	CD36 molecule	Plasma Membrane	-1.6
**CXCL5**	chemokine (C-X-C motif) ligand 5	Extracellular Space	-1.6
**MAP3K14**	mitogen-activated protein kinase kinase kinase 14	Cytoplasm	-1.74
**CCL7**	chemokine (C-C motif) ligand 7	Extracellular Space	-1.78
**IL15RA**	interleukin 15 receptor subunit alpha	Plasma Membrane	-1.81
**NFKB1**	nuclear factor of kappa light polypeptide gene enhancer in B-cells 1	Nucleus	-1.82
**CXCL2**	chemokine (C-X-C motif) ligand 2	Extracellular Space	-1.88
**TNFSF4**	tumor necrosis factor superfamily member 4	Extracellular Space	-1.93
**IL10RB**	interleukin 10 receptor subunit beta	Plasma Membrane	-2.1
**CCL8**	chemokine (C-C motif) ligand 8	Extracellular Space	-2.14
**CCL2**	chemokine (C-C motif) ligand 2	Extracellular Space	-3.47
**IL2RA**	interleukin 2 receptor subunit alpha	Plasma Membrane	-7.02
**IL12B**	interleukin 12B	Extracellular Space	-11.81

Supplementary figures legend

Supplementary Fig.1

A: relative expression of miR-29b in immature dendritic cells (iDCs) as compared to monocytes.

B: evaluation of miR-29b transfection efficiency in DCs through qRT-PCR after enforced expression trough electroporation.

C: Unsupervised Hierarchical clustering of gene expression profiling performed on dendritic cells from three different healthy donors transiently transfected with a negative control (NC) or miR-29b mimics and co-cultured with U266 MM cell line for 24 hours.

D shows the main Canonical Pathways modulated by miR-29b enforced expression in dendritic cells according to Ingenuity Pathway Analysis.

E reports the "Dendritic Cell Maturation" canonical pathway, highlighting genes downregulated (green) or upregulated (red) as well as signal paths predicted to be inhibited (blue) or activated (orange) by miR-29b enforced expression in dendritic cells.

F: main signaling networks involving cell movement and chemotaxis processes according to Ingenuity Pathway Analysis modulated by miR-29b enforced expression in dendritic cells.

Supplementary Fig.2

A reports the "inflammatory/immunologic mediators" network obtained from the merging of the previously selected networks involving cell movement and chemotaxis processes. This picture underscores genes downregulated (green) or upregulated (red) as well as signal paths predicted to be inhibited (blue) or activated (orange) by miR-29b enforced expression in dendritic cells.

B: comparative analysis of the main functions modulated in inflammatory dendritic cells and miR-29 transfected dendritic cells as compared to their respective control (blue: inhibition of function and orange: activation of function). On the right, fold changes relative to all genes involved in "Dendritic Cell Maturation" canonical pathway "inflammatory/immunologic mediators" network for both inflammatory dendritic cells or miR-29b transfected dendritic cells.

C: validation through qRT-PCR of deregulation of identified in gene expression analysis.

Supplementary Fig.3

A: comparison of expression of CD86 (surface expression, median fluorescence intensity, MFI) and B7H3 (both MFI and protein level) in dendritic cells after miR-29b enforced expression and 48h co-culture with MM cell line (U266).

B: protein levels of JUN and MAP2K4 in dendritic cells co-cultured with a second MM cell line (RPMI-8226) after miR-29b enforced expression.

C: evaluation of the effect of miR-29b transient overexpression in DCs co-cultured for 48h with U266 MM cell line on MCL1 protein level

D: results from tubulogenic assay performed in the presence of supernatant from 29b-DCs or NC-DCs co-cultured with U266 MM cell lines. Images have been analyzed with ImageJ software and Angiogenesis analyzer plugin. Legend: red points surrounded by blue = nodes (pixels that have at least 3 neighboring elements corresponding to a bifurcation) surrounded by junctions symbol; red surrounded by yellow = extremities; green = branches (elements constituted by a junction and one extremity); magenta = segments (elements between two junctions); orange = master segments (segments where none of the two junctions is implicated with one branch); blue sky = meshes (areas enclosed by segments or master segments, they are made by tube-like-structure); junctions surrounded by red = master junctions (junctions linking at least three master segments); blue and cyan = isolated elements. *: $p<0.05$

E: predicted modulation by Ingenuity Pathway Analysis and subsequent validation at protein level of inflammasome pathway, induced by miR-29b enforced expression in dendritic cells co-cultured for 48h with MM cells.

F: effects of miR-29b transient transfection on STAT1 phosphorylation in dendritic cells co-cultured with 2 different MM cell lines (AMO and U266)

G: predicted effects induced by miR-29b on AKT pathway.

Supplementary Fig.4

A: Evaluation of the capability of DCs to induce Th17 (IL17+ lymphocytes) expansion in presence or absence of U266 MM cells.

B: histograms reporting the average increase in the presence of CD3+/IL17a+ in lymphocytes co-cultured with MM educated DCs.

C: representative plots of lymphocytes co-cultured with RPMI8226-educated DCs or DCs alone where there is a relevant increase in CD3+/IL17a+ lymphocytes in the presence of MM cells.

D: evaluation of tumor proliferation and angiogenesis after NC or miR-29b treatment through Ki67 and CD31 evaluation by immunohistochemistry on scaffold retrieved from SCID-synth-hu models.

Supplementary Fig.5

A: dot-plots of expression of cytokine and chemokine receptors whose ligands have been demonstrated to be modulated by miR-29b enforced expression in DCs. Each comparison have been made against healthy donors (HD). *:$p<0.05$

B: Cox regression multivariate analysis results where the independent prognostic value of each of the chemokine receptor genes (dichotomized on the median value) was evaluated in the presence of known prognostic factors (ISS and R-ISS) . Results are reported accordingly to survival outcome measure analyzed (PFS or OS) and confounding factor introduced in the analysis (ISS or R-ISS).

C: protein levels of cleaved PARP, total PARP and BCL2 in MM cells after 48h co-culture with DCs transfected with miR-29b mimics.

Supplementary Fig. 1

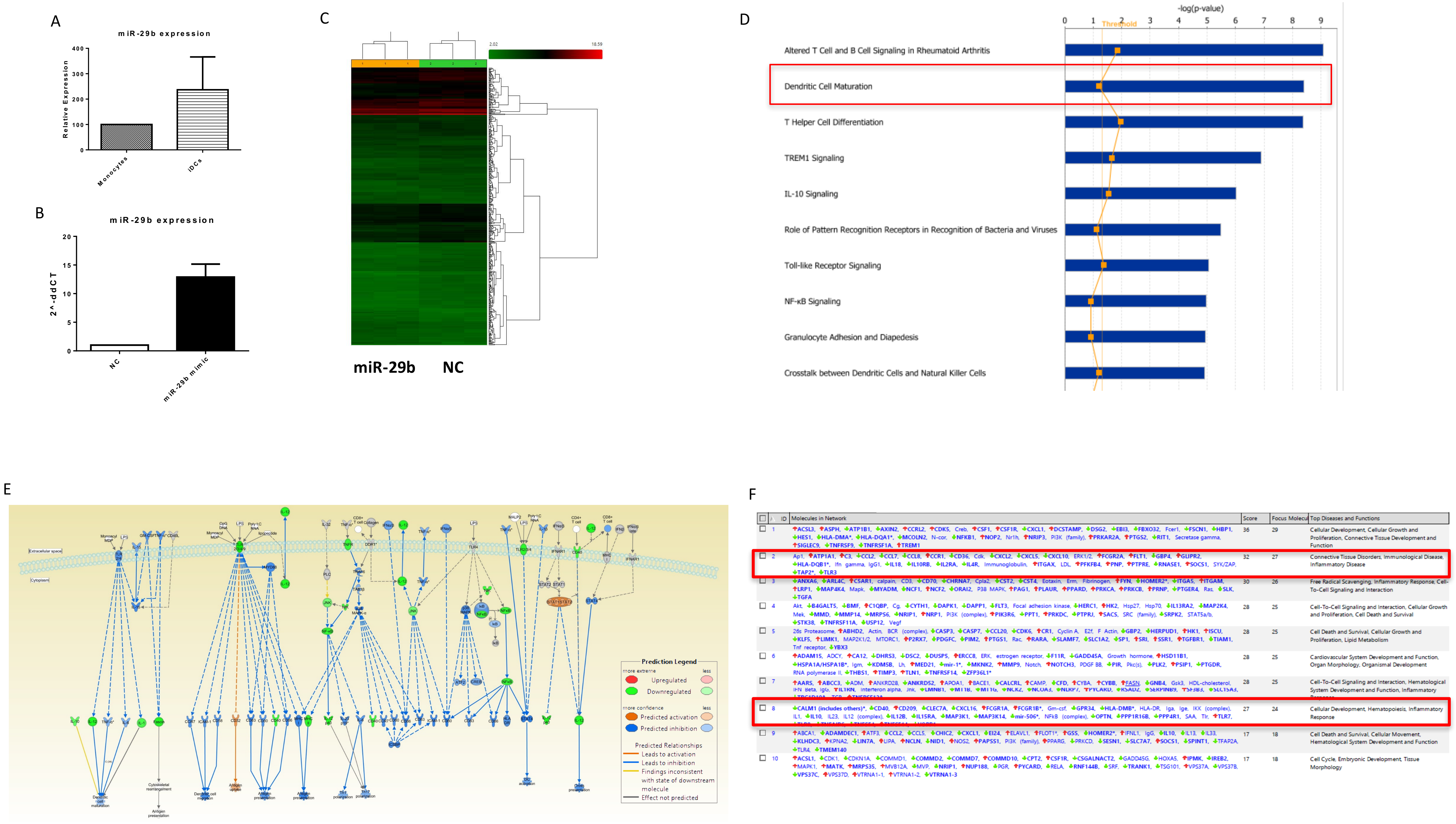

Supplementary figure 2

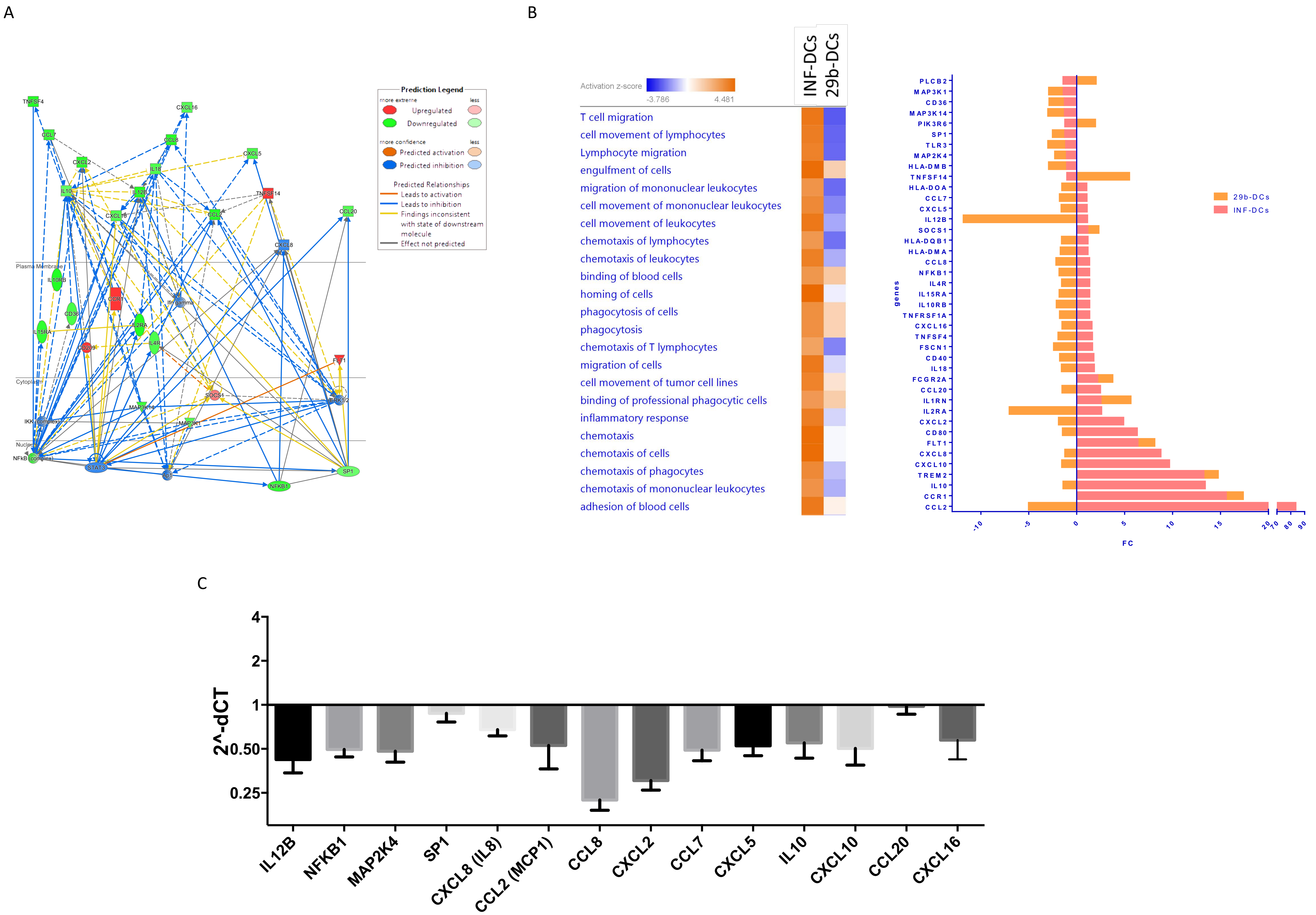

Supplementary figure 3

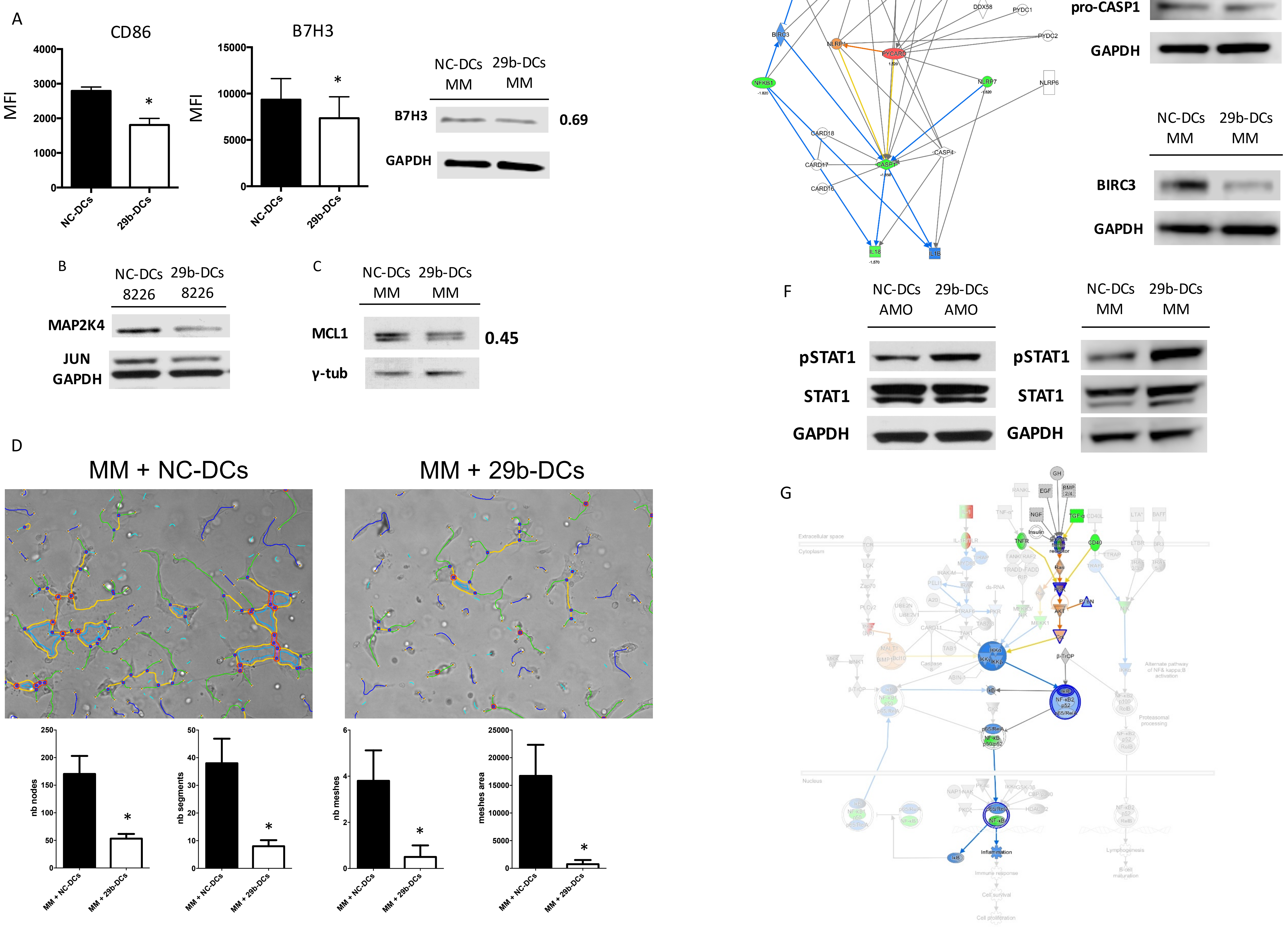

Supplementary figure 4

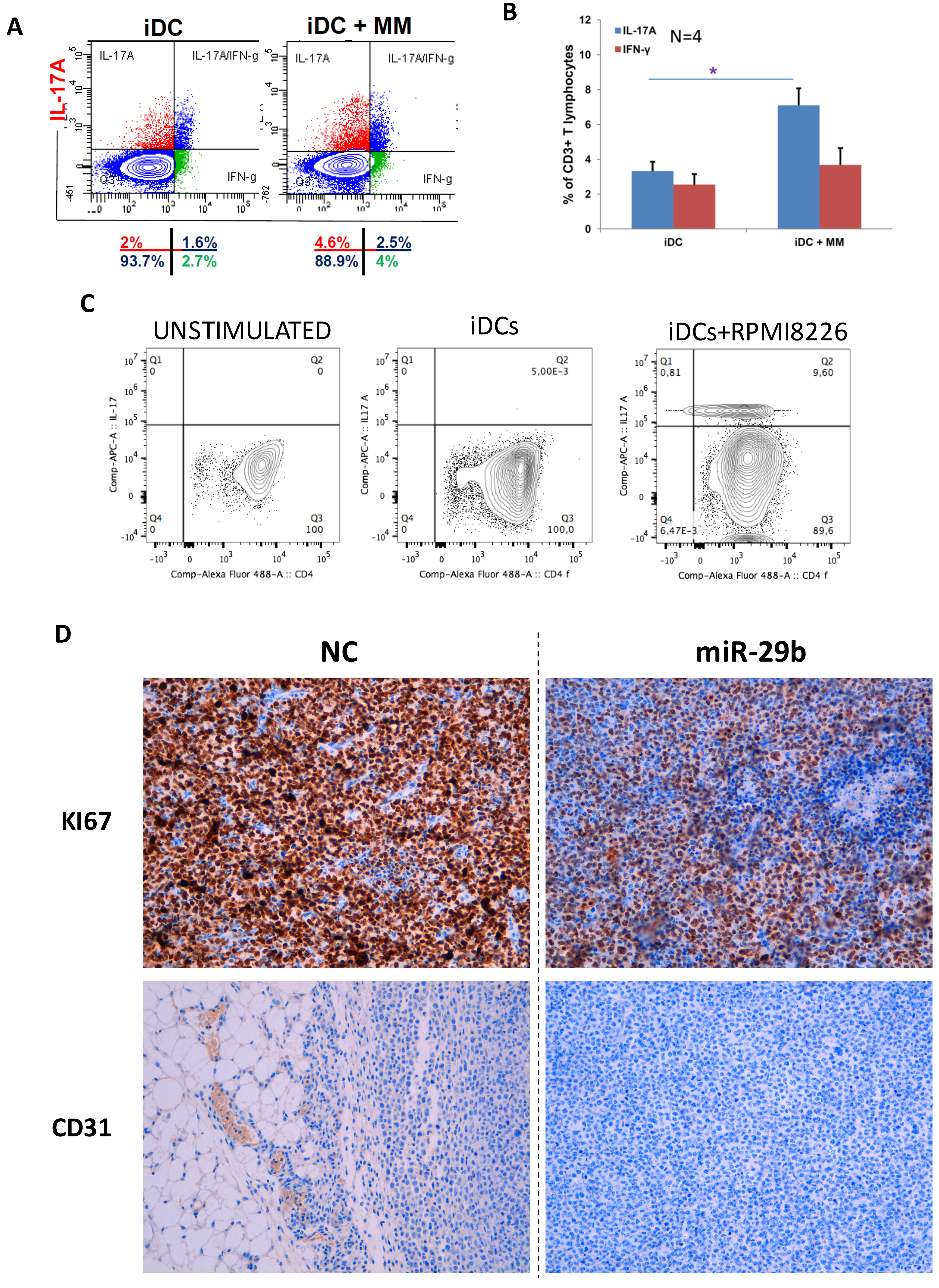

Supplementary figure 5

A

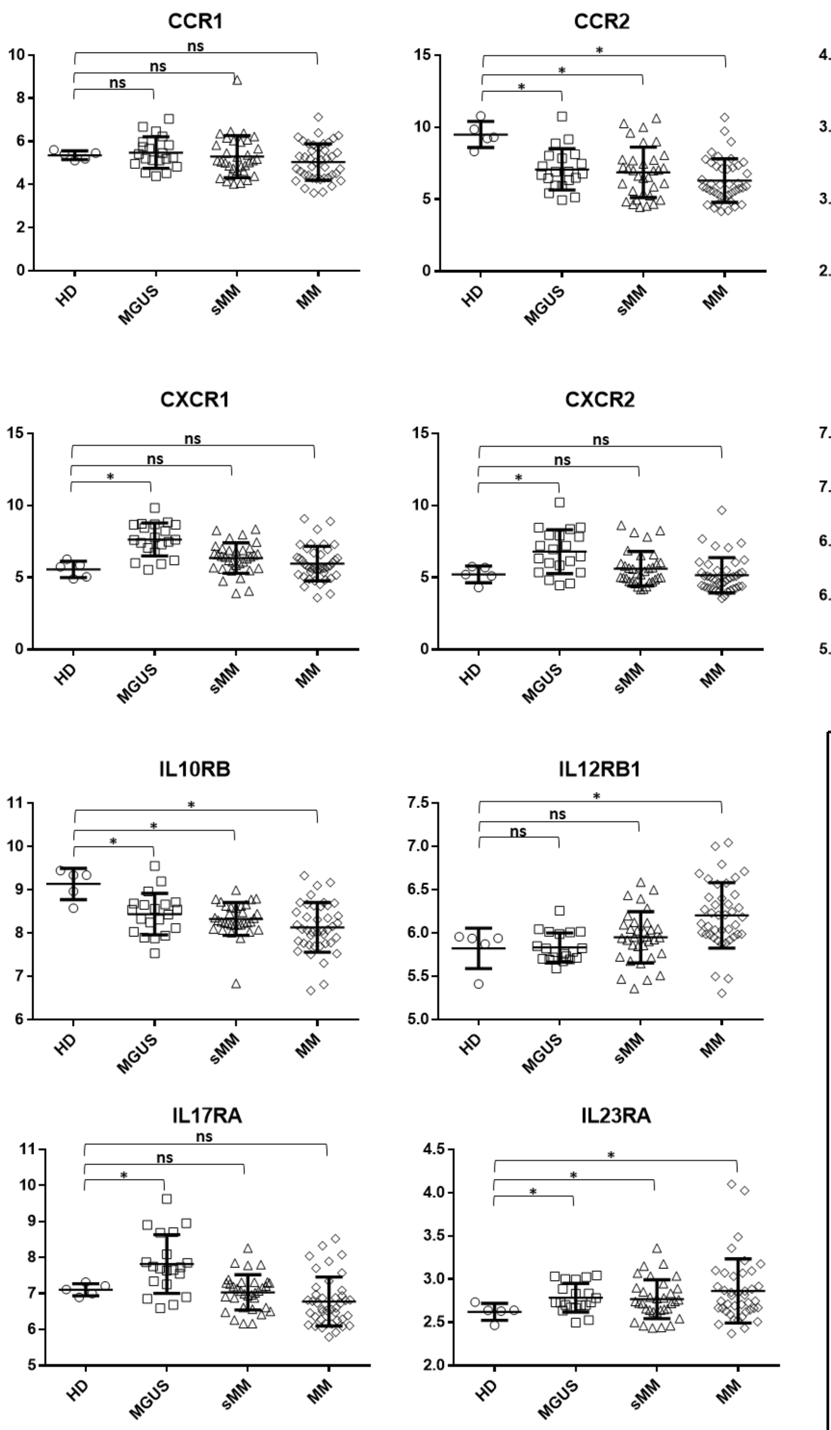

B

Cox regression analyses

ISS

PFS

	p value	HR	95.0% CI for HR	
			Lower	Upper
**ISS**	**.000**	**1.555**	**1.221**	**1.979**
**IL17RA**	**.004**	**1.822**	**1.212**	**2.739**
**CXCR6**	**.038**	**1.530**	**1.023**	**2.288**
CCR5	.057	.619	.378	1.015
CCR4	.118	1.371	.923	2.035
CXCR1	.165	1.481	.850	2.578
CXCR3	.233	1.283	.852	1.934
CCR3	.251	1.319	.822	2.117
IL12RB1	.291	.805	.538	1.204
CXCR2	.355	.767	.437	1.345
IL23R	.509	1.141	.771	1.689
CCR1	.561	1.149	.719	1.835
CCR2	.622	.896	.579	1.386
IL10RA	.629	1.110	.727	1.693
IL10RB	.697	.925	.624	1.371

OS

	p value	HR	95.0% CI for HR	
			Lower	Upper
**ISS**	**.000**	**2.456**	**1.676**	**3.599**
**CCR3**	**.007**	**2.552**	**1.289**	**5.053**
**IL17RA**	**.045**	**1.841**	**1.014**	**3.343**
CCR5	.057	.523	.268	1.021
CXCR3	.107	1.613	.901	2.886
CCR4	.216	1.423	.814	2.488
CXCR2	.238	.611	.269	1.385
CCR1	.244	1.466	.770	2.790
CXCR1	.313	1.514	.677	3.388
CXCR6	.320	1.336	.755	2.366
IL12RB1	.517	.828	.469	1.463
IL10RB	.580	.852	.483	1.503
IL23R	.597	1.161	.669	2.014
IL10RA	.759	.910	.499	1.659
CCR2	.896	.960	.520	1.772

R-ISS

PFS

	p value	HR	95.0% CI for HR	
			Lower	Upper
**IL17RA**	**.006**	**1.954**	**1.209**	**3.156**
**R_ISS**	**.008**	**1.742**	**1.159**	**2.619**
IL23R	.194	1.356	.856	2.149
CCR4	.195	1.347	.858	2.114
CXCR6	.242	1.329	.825	2.141
CCR5	.392	.778	.439	1.381
CCR2	.412	.811	.491	1.338
IL12RB1	.448	.833	.520	1.335
IL10RB	.454	.840	.532	1.326
CCR3	.519	1.201	.688	2.098
IL10RA	.563	1.155	.709	1.880
CXCR1	.615	1.171	.632	2.170
CXCR2	.694	.881	.469	1.655
CCR1	.822	1.064	.617	1.836
CXCR3	.837	1.051	.654	1.689

OS

	p value	HR	95.0% CI for HR	
			Lower	Upper
**R_ISS**	**.002**	**2.510**	**1.386**	**4.547**
**IL17RA**	**.019**	**2.261**	**1.141**	**4.481**
**CCR3**	**.048**	**2.226**	**1.006**	**4.926**
CCR4	.226	1.483	.784	2.804
IL10RA	.381	.736	.371	1.460
CXCR3	.478	1.272	.654	2.473
CXCR2	.481	.720	.289	1.797
IL23R	.507	1.241	.656	2.346
CXCR6	.515	1.251	.637	2.456
IL10RB	.558	.824	.431	1.575
CCR5	.562	.797	.371	1.714
CXCR1	.679	1.208	.493	2.960
CCR1	.741	1.134	.537	2.394
IL12RB1	.979	.991	.514	1.911
CCR2	.981	.992	.496	1.983

C

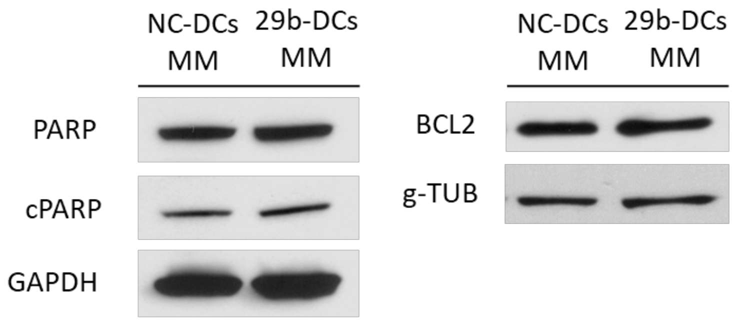

Supplement: Supplementary Data [file leu2017336x1.pdf]
